# The Comparison of Short- and Long-Term Outcomes for Laparoscopic Versus Open Gastrectomy for Patients With Advanced Gastric Cancer: A Meta-Analysis of Randomized Controlled Trials

**DOI:** 10.3389/fonc.2022.844803

**Published:** 2022-04-05

**Authors:** Jinyan Jiang, Guanxiong Ye, Jun Wang, Xiaoya Xu, Kai Zhang, Shi Wang

**Affiliations:** ^1^ Department of Nursing, Lishui People’s Hospital, Lishui, China; ^2^ Department of General Surgery, Lishui People’s Hospital, Lishui, China; ^3^ Department of Critical Care Medicine, Second Affiliated Hospital, Zhejiang University School of Medicine, Hangzhou, China

**Keywords:** laparoscopic gastrectomy (LG), open gastrectomy (OG), advanced gastric cancer (AGC), postoperative complication, survival rate (SR), meta-analysis, randomized controlled trial

## Abstract

**Objectives:**

The effect of laparoscopic gastrectomy (LG) for the treatment of advanced gastric cancer (AGC) is still controversial. The aim of this meta-analysis was to contrast the short- and long-term outcomes of laparoscopic versus conventional open gastrectomy (OG) for patients with AGC.

**Methods:**

Databases including PubMed, Embase, Scopus, and Cochrane Library were systematically searched until December 2021 for randomized controlled trial-enrolled patients undergoing LG or OG for the treatment of AGC. Short-term outcomes were overall postoperative complications, anastomotic leakage, number of retrieved lymph node, surgical time, blood loss, length of hospital stay, and short-term mortality. Long-term outcomes were survival rates at 1, 3, and 5 years.

**Results:**

A total of 12 trials involving 4,101 patients (2,059 in LG group, 2,042 in OG group) were included. No effect on overall postoperative complications (OR 0.84, 95% CI 0.67 to 1.05, p = 0.12, I^2^ = 34%) and anastomotic leakage (OR 1.26, 95% CI 0.82 to 1.95, p = 0.30, I^2^ = 0%) was found. Compared with the open approach, patients receiving LG had fewer blood loss (MD -54.38, 95% CI -78.09 to -30.67, p < 0.00001, I^2^ = 90%) and shorter length of hospital stay (MD -1.25, 95% CI -2.08 to -0.42, p = 0.003, I^2^ = 86%). However, the LG was associated with a lower number of retrieved lymph nodes (MD -1.02, 95% CI -1.77 to -0.27, p = 0.008, I^2^ = 0%) and longer surgical time (MD 40.87, 95% CI 20.37 to 54.44, p < 0.00001, I^2^ = 94%). Furthermore, there were no differences between LG and OG groups in short-term mortality and survival rate at 1, 3, and 5 years.

**Conclusions:**

LG offers improved short-term outcomes including shorter hospital stays and fewer blood loss, with comparable postoperative complications, short-term mortality, and survival rate at 1, 3, and 5 years when compared to the open approach. Our results support the implementation of LG in patients with AGC.

**Systematic Review Registration:**

PROSPERO (CRD 42021297141).

## Introduction

Gastric cancer is one of the most common cancers and a main economic burden worldwide (1). According to the GLOBOCAN 2020 data, gastric cancer is the fifth most common malignancy and the fourth leading cause of cancer death, causing an estimated 768,793 deaths in 2020 globally (2). Surgical resection with lymphadenectomy is the cornerstone of multimodality curative treatment, and open gastrectomy (OG) has long been the gold standard worldwide (3). However, since Kitano et al. (4) reported the first laparoscopic gastrectomy (LG) for the treatment of early-stage distal gastric cancer in 1994, this minimally invasive technique has been rapidly developed and in the field of gastric cancer, especially for treatment of early gastric cancer (EGC) (5, 6).

Nowadays, the LG has gained growing popularity in the treatment of EGC since this minimally invasive technique has some definite benefits including lower postoperative complications, faster recovery, shortened postoperative length of stay, and better quality of life. Previously, several well-designed randomized controlled trials (RCTs) from China, Korea, and Japan demonstrated the beneficial short-term outcomes of laparoscopic distal gastrectomy (LDG) including less blood loss and postoperative pain, faster recovery, and shorter hospital stay, and similar oncologic safety to the open approach (7–10). However, despite the extensive use of laparoscopic surgery, whether this minimally invasive approach is beneficial for patients with advanced gastric cancer (AGC) remains controversial.

Recently, the CLASS-01 (11) and KLASS-02 trials (12) updated their results of long-term outcomes, indicating that locally AGC patients with LDG had similar long-term survival rates compared to open distal gastrectomy. Moreover, the LOGICA trial (13), which compared the LG with OG for treatment of AGC in the Western population, reported comparable outcomes including postoperative complications, length of hospital stay, R0 resection rate, lymph node yield, and 1-year overall survival (OS) rate. Therefore, in order to summarize the current high-quality evidence, we performed this meta-analysis of RCTs to compared the short- and long-term outcomes of LG versus OG for patients with AGC.

## Methods

We conducted this meta-analysis according to the updated PRISRMA statement (14) (**Supplementary Material 1**) and registered the protocol on PROSPERO (CRD 42021297141). A literature search was performed in PubMed, Embase, Scopus, and Cochrane Library for eligible RCTs in English from inception through December 2021. The search used broad search terms containing “gastric cancer,” “gastric carcinoma,” “stomach cancer,” “laparoscopic,” “laparoscopy,” “open gastrectomy,” and “RCT” (complete search strategies in **Supplementary Material 2**).

### Eligibility Criteria

The inclusion criteria were as follows: 1) population: adult patients (older than 18 years) with AGC; 2) intervention: laparoscopic surgery for gastrectomy; 3) comparison: open surgery for gastrectomy; 4) outcomes: short-term outcomes including postoperative complication, number of retrieved lymph nodes, surgical time, blood loss, length of hospital stay, and short-term mortality (including in-hospital mortality, or mortality within 30 days after operation). Long-term outcomes were survival rate at 1, 3, and 5 years, including OS rate and disease-free survival (DFS) rate; 5) design: RCT.

### Data Extraction and Quality Assessment

The data from included trials were independently extracted by two reviewers (JJ and SW). The characteristics of included studies (e.g., study, years of publication, study location, population, number of patients, intervention, outcomes) are recorded in **Table 1**.

**Table 1:** Characteristics of included studies.

Study	Study period, location	Population	Intervention and number of patients	Outcomes
CLASS-01 Trial (11, 15, 16)	September 2012 to December 2014, in China	Patients with cT2-4aN0-3M0 gastric cancer to undergo either LDG or ODG with D2 lymphadenectomy	LDG (n = 519), ODG (n = 520)	Short-term outcomes: hospital stay, operative time, number of retrieved lymph node, blood loss, postoperative complications, in-hospital mortality Long-term outcomes: OS rate at 1, 3, and 5 years
KLASS-02 Trial (12, 17)	November 2011 to April 2015, in Korea	Patients with cT2-4aN0-3M0 gastric carcinoma to undergo either LDG or ODG with D2 lymphadenectomy	LDG (n = 513), ODG (n = 498)	Short-term outcomes: hospital stay, operative time, number of retrieved lymph node, blood loss, postoperative complications, 90-day mortality Long-term outcomes: DFS rate at 1 and 3 years
LOGICA Trial (13)	February 2015 to August 2018, in Netherlands	Patients with gastric adenocarcinoma (cT1-4aN0-3bM0) to undergo total or distal gastrectomy with total omentectomy and D2 lymphadenectomy	LDG/LTG (n = 115), ODG/OTG (n = 110)	Short-term outcomes: hospital stay, operative time, number of retrieved lymph node, blood loss, postoperative complications, in-hospital mortality Long-term outcomes: OS rate at 1 year
Luo et al. (18, 19)	May 2008 to April 2012, in China	Patients with cT2-4N0-3M0 gastric cancer, and could undergo D2 resection	Hand-assisted LDG (n = 62), ODG (n = 62)	Short-term outcomes: hospital stay, operative time, number of retrieved lymph node, blood loss, postoperative complications, in-hospital mortality Long-term outcomes: OS and FDS rate at 1, 3, and 5 years
Li et al. (20)	April 2015 to November 2017, in China	Patients with locally advanced gastric cancer (cT2-4aN0-3M0) to either LDG or ODG with D2 lymphadenectomy	LDG (n = 45), ODG (n = 50)	Short-term outcomes: hospital stay, operative time, number of retrieved lymph node, blood loss, postoperative complications, in-hospital mortality
Shi et al. (21, 22)	January 2010 to June 2012, in China	Patients with cT2-3N0-3M0 gastric cancer to LAG or OG with D2 lymphadenectomy	LDG/LTG (n = 162), ODG/OTG (n = 160)	Short-term outcomes: hospital stay, operative time, number of retrieved lymph node, blood loss, postoperative complications, in-hospital mortality Long-term outcomes: OS and DFS rate at 1, 3, and 5 years
Wang et al. (23)	March 2014 to August 2017, in China	Patients with gastric cancer (cT2-4aN0-3M0) to either LDG or ODG with D2 lymphadenectomy	LDG (n = 222), ODG (n = 220)	Short-term outcomes: hospital stay, operative time, number of retrieved lymph node, blood loss, postoperative complications, in-hospital mortality
Guo et al. (24)	December 2016 to December 2017, in China	Patients with gastric cancer (cT2-4aN0-3M0) to either LTG or OTG with D2 lymphadenectomy	LTG (n = 114), OTG (n = 108)	Short-term outcomes: hospital stay, operative time, number of retrieved lymph node, blood loss, postoperative complications, in-hospital mortality
COACT 1001 trial (25)	June 2010 to October 2011, in Korea	Patients with gastric cancer (cT2-4aN0-2M0) to either LDG or ODG with D2 lymph node dissection	LDG (n=100), ODG (n=96)	Short-term outcomes: hospital stay, operative time, number of retrieved lymph node, blood loss, postoperative complications Long-term outcomes: DFS rate at 1, 3 year
Cui et al. (26)	October 2010 to September 2012, in China	Patients with adenocarcinoma of stomach with no distant metastases, to undergo either LG or OG with D2 lymphadenectomy	LDG/LTG (n = 128), ODG/OTG (n = 142)	Short-term outcomes: hospital stay, operative time, number of retrieved lymph node, blood loss, postoperative complications, in-hospital mortality
Cai et al. (27)	March 2008 to December 2009, in China	Patients with advanced gastric cancer to either LG or OG with D2 lymphadenectomy	LDG (n = 49), ODG (n = 47)	Short-term outcomes: hospital stay, operative time, number of retrieved lymph node, blood loss, postoperative complications Long-term outcomes: OS rate at 3 year
Huscher et al. (28)	November 1992 to February 1996, in Italy	Patients with a preoperative diagnosis of distal gastric cancer to either LTG or OTG with D1 or D2 lymphadenectomy	LTG (n = 30), OTG (n = 29)	Short-term outcomes: hospital stay, operative time, number of retrieved lymph node, blood loss, postoperative complications Long-term outcomes: OS and DFS rate at 5 year

LDG, laparoscopic distal gastrectomy; ODG, open distal gastrectomy; LTG, laparoscopic total gastrectomy; OTG, open total gastrectomy; LAG, laparoscopic-assisted gastrectomy; OS, overall survival; DFS, disease-free survival.

For the methodological quality of including studies, two authors (JJ and SW) independently assessed the quality by using the Cochrane risk-of-bias tool (29).

### Statistical Synthesis and Analysis

For short-term outcomes, we combined data from included studies to estimate the pooled odds ratio (OR) with 95% confidence interval (CI) for dichotomous outcomes, and continuous outcomes were pooled as mean difference (MD) with 95% CI. The meta-analysis of OS and DFS used the hazard ratio (HR) with 95% CI reported in the primary studies. If the primary studies did not provide the HR data, we obtained the HR data by digitizing the Kaplan–Meier survival curves (30). The heterogeneity between studies was tested by the chi-squared test with significance set at p value of 0.1, and quantitatively by inconsistency (I^2^) statistics (31). Substantial heterogeneity was identified when I^2^ value >30%, and we employed a random-effect model to perform the analysis; otherwise, a fixed-effect model would be used. In addition, we used the funnel plot and Egger’s regression test to assess the publication bias (32).

A predefined subgroup analysis was performed based on the extent of resection (partial versus total gastrectomy) and tumor stage (clinical stage II versus stage III). In addition, a sensitivity analysis by omitting each one trial at a time was performed to explore the effect of individual trials.

## Results

### Study Identification and Characteristics

**Figure 1:**
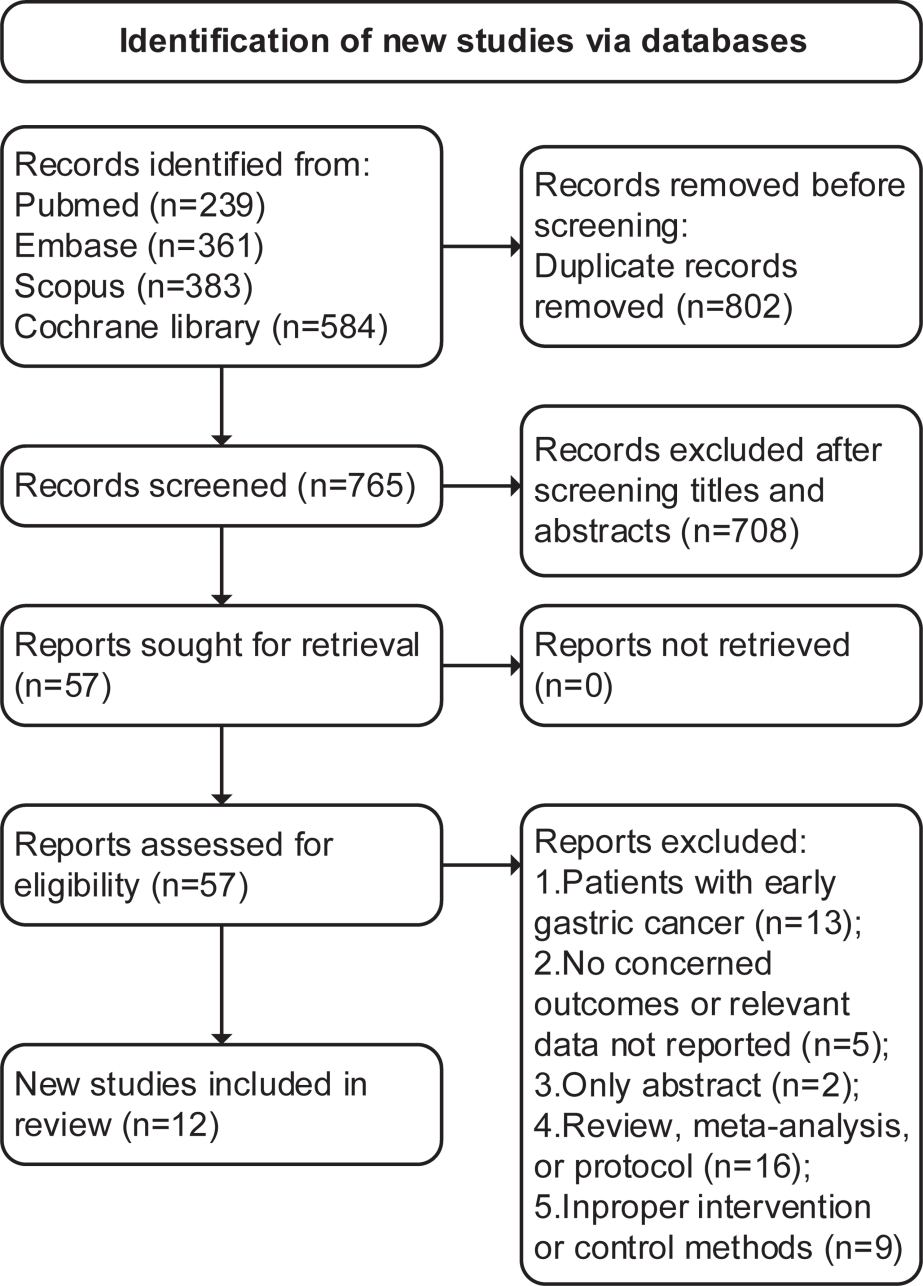
PRISMA 2020 flow diagram for the meta-analysis.

The initial search identified 1,567 articles (239 from PubMed, 361 from Embase, 383 from Scopus, and 584 from Cochrane Library), 802 were duplications, and 708 studies were excluded through title and abstract screening. In the full-text assessments, 45 studies were further excluded with reasons and a total of 12 trials with 17 articles (11–13, 15–28) were finally included (search process in **Figure 1**).

**Table 1** presents the characteristics of including trials. A total of 4,101 patients with ACG were analyzed, 2,059 in the LG group and 2,042 in the OG group. The sample size of included trials ranged from a minimum of 59 up to 1,039. Among the 12 included trials, eight were done in China (11, 15, 16, 18–24, 26, 27), two in Korea (12, 17, 25), one in Netherlands (13), and one in Italy (28). In each included trial, the LG and OG groups were similar as regards age, gender, tumor size, clinical TNM stage, and the American Society of Anesthesiologist (ASA) score. The types of operation varied among each trial, seven trials (11, 12, 15–20, 23, 25, 27) performed partial gastrectomy, and two (24, 28) performed total gastrectomy; the operation in the rest of the three trials (13, 21, 22, 26) included total or partial gastrectomy.

In addition, the number of retrieved lymph nodes, length of hospital stay, and blood loss in two trials (13, 20) were expressed in the form of median and interquartile range (IQR). Thus, we used the methodology of Wan et al. (33) to convert these data into mean and standard deviation (SD).

### Quality Assessment

**Figure 2:**
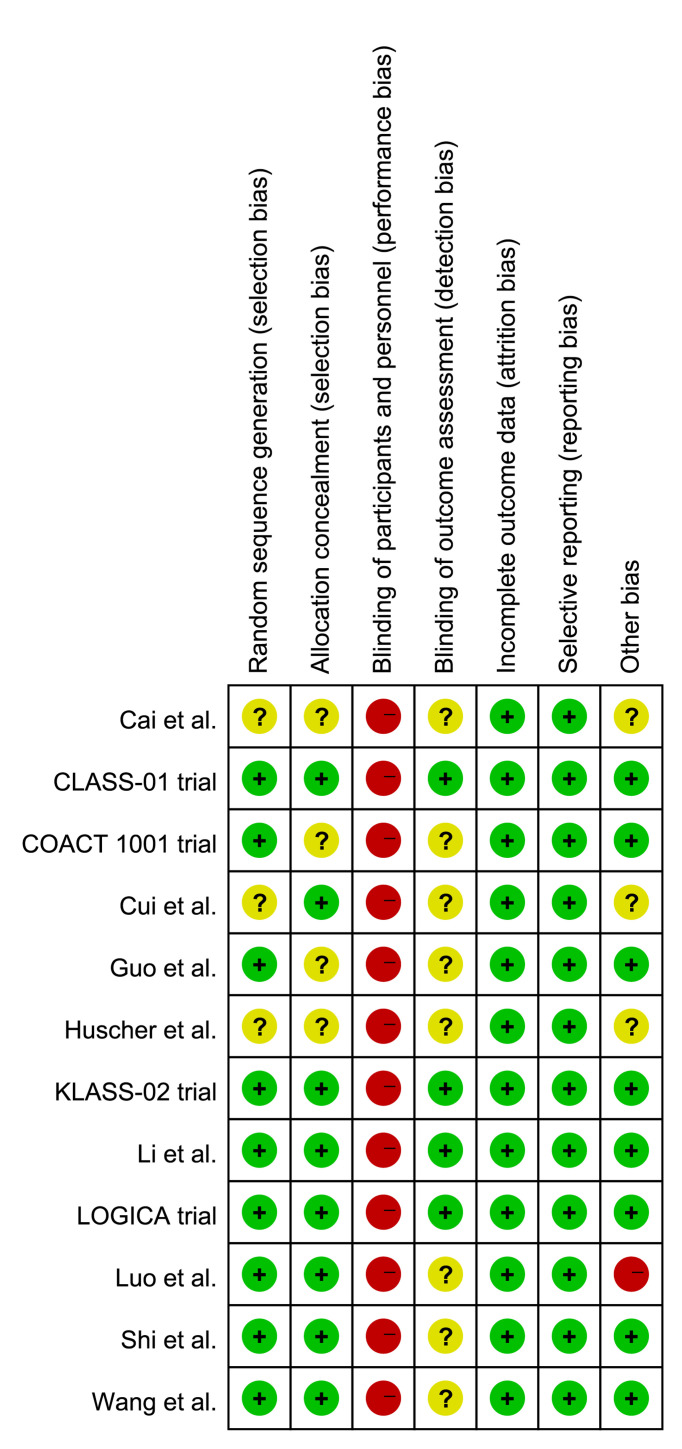
Assessment of quality by the Cochrane risk of bias tool.

The quality assessment results are presented in **Figure 2**. Since all included trials were open-label study, they had a high risk of performance bias. Three trials (26–28) did not provide the detailed information of random sequence generation and four trials (24, 25, 27, 28) did not state the allocation concealment. The blinding method for outcome assessment was unclear in eight trials (19, 22–28). Moreover, Cai et al. (27), Cui et al. (26), and Huscher et al. (28) did not clarify the clinical TMN stage of included patients in the inclusion criteria, and Luo et al. performed a hand-assisted laparoscopic surgery, which was different from other trials.

The funnel plot and Egger’s test were used to evaluate the publication bias (**Supplementary Material 3**); the results showed that there was potential risk of publication bias for the blood loss and 5-year survival rate (Egger’s test, p < 0.10). Therefore, we used the trim-and-fill method to perform an additional analysis. The analysis after imputing continued to show that the LG group was associated with decreased blood loss (MD -37.13, 95% CI -62.20 to -12.06, p = 0.0037, I^2^ = 91%) and similar 5-year survival rate (OR 0.85, 95% CI 0.69 to 1.04, p = 0.12, I^2^ = 0%).

### Short-Term Outcomes

**Figure 3:**
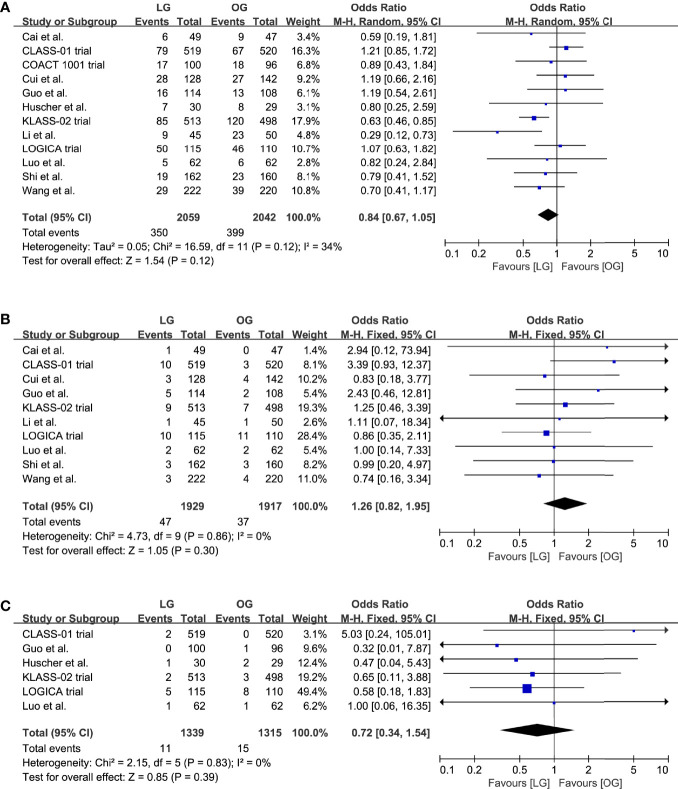
Pooled estimates of **(A)** overall postoperative complications, **(B)** incidence of anastomotic leakage, **(C)** short-term mortality.

All included trials reported the incidence of overall postoperative complications. The pooled analysis indicated no effect on the overall postoperative complications (OR 0.84, 95% CI 0.67 to 1.05, p = 0.12, I^2^ = 34%; **Table 2**, **Figure 3A**). Moreover, we compared the incidence of anastomotic leakage; the results indicated that there was no significant difference of anastomotic leakage rate between the two surgical options (OR 1.26, 95% CI 0.82 to 1.95, p = 0.30, I^2^ = 0%; **Table 2**, **Figure 3B**).

**Table 2:** Results of this meta-analysis.

Outcome	N	Result (laparoscopic versus open gastrectomy)
**Short-term outcomes**		
Postoperative complications	12	OR 0.84, 95% CI 0.67 to 1.05, p = 0.12, I^2^ = 34%
Partial gastrectomy	7	OR 0.73, 95% CI 0.52 to 1.02, p = 0.07, I^2^ = 54%
Total gastrectomy	2	OR 1.05, 95% CI 0.55 to 2.02, p = 0.87, I^2^ = 0%
Partial/total gastrectomy	3	OR 1.02, 95% CI 0.73 to 1.43, p = 0.90, I^2^ = 0%
		Test for subgroup difference: I^2^ = 9%
Minor complications	12	OR 0.87, 95% CI 0.73 to 1.05, p = 0.14, I^2^ = 27%
Major complications	12	OR 0.87, 95% CI 0.67 to 1.14, p = 0.32, I^2^ = 0%
		Test for subgroup difference: I^2^ = 0%
Anastomotic leakage	10	OR 1.26, 95% CI 0.82 to 1.95, p = 0.30, I^2^ = 0%
Partial gastrectomy	6	OR 1.51, 95% CI 0.82 to 2.78, p = 0.18, I^2^ = 0%
Total gastrectomy	1	OR 2.43, 95% CI 0.46 to 12.81, p = 0.29
Partial/total gastrectomy	3	OR 0.87, 95% CI 0.43 to 1.75, p = 0.70, I^2^ = 0%
		Test for subgroup difference: I^2^ = 0%
Short-term mortality	6	OR 0.72, 95% CI 0.34 to 1.54, p = 0.39, I^2^ = 0%
Partial gastrectomy	3	OR 1.21, 95% CI 0.35 to 4.20, p = 0.77, I^2^ = 0%
Total gastrectomy	2	OR 0.40, 95% CI 0.06 to 2.80, p = 0.36, I^2^ = 0%
Partial/total gastrectomy	1	OR 0.58, 95% CI 0.18 to 1.83, p = 0.35
		Test for subgroup difference: I^2^ = 0%
Length of hospital stay	11	MD -1.25, 95% CI -2.08 to -0.42, p = 0.003, I^2^ = 86%
Partial gastrectomy	6	MD -0.59, 95% CI -1.12 to -0.07, p = 0.03, I^2^ = 43%
Total gastrectomy	2	MD -2.94, 95% CI -5.29 to -0.59, p = 0.01, I^2^ = 65%
Partial/total gastrectomy	3	MD -2.11, 95% CI -4.53 to 0.32, p = 0.09, I^2^ = 94%
		Test for subgroup difference: I^2^ = 59%
Blood loss	11	MD -54.38, 95% CI -78.09 to -30.67, p < 0.00001, I^2^ = 90%
Partial gastrectomy	6	MD -31.97, 95% CI -50.42 to -13.53, p = 0.0007, I^2^ = 70%
Total gastrectomy	2	MD -87.21, 95% CI -225.86 to 51.44, p = 0.22, I^2^ = 92%
Partial/total gastrectomy	3	MD -87.77, 95% CI -146.90 to -28.63, p < 0.00001, I^2^ = 94%
		Test for subgroup difference: I^2^ = 45%
Surgical time	4	MD 40.87, 95% CI 27.31 to 54.44, p < 0.00001, I^2^ = 94%
Partial gastrectomy	3	MD 46.22, 95% CI 25.90 to 66.55, p < 0.00001, I^2^ = 96%
Total gastrectomy	1	MD 20.79, 95% CI 4.24 to 37.34, p = 0.01, I^2^ = 54%
Partial/total gastrectomy		MD 42.41, 95% CI 22.47 to 62.35, p < 0.0001, I^2^ = 83%
		Test for subgroup difference: I^2^ = 56%
Number of retrieved LN	12	(MD -1.02, 95% CI -1.77 to -0.27, p = 0.008, I^2^ = 0%)
Partial gastrectomy	7	(MD -1.32, 95% CI -2.37 to -0.27, p = 0.01, I^2^ = 0%)
Total gastrectomy	2	(MD -1.33, 95% CI -5.76 to 3.10, p = 0.56, I^2^ = 0%)
Partial/total gastrectomy	3	(MD -0.67, 95% CI -1.78 to 0.44, p = 0.24, I^2^ = 0%)
		Test for subgroup difference: I^2^ = 0%
**Long-term outcomes**		
1-year survival rate	7	HR 1.11, 95% CI 0.80 to 1.56, p = 0.53, I^2^ = 0%
Partial gastrectomy	4	HR 1.09, 95% CI 0.67 to 1.79, p = 0.72, I^2^ = 0%
Total gastrectomy	1	HR 0.97, 95% CI 0.16 to 5.98, p = 0.97
Partial/total gastrectomy	2	HR 1.14, 95% CI 0.71 to 1.84, p = 0.58, I^2^ = 0%
		Test for subgroup difference: I^2^ = 0%
<Stage III	5	HR 0.99, 95% CI 0.51 to 1.90, p = 0.97, I^2^ = 0%
≥Stage III	5	HR 1.16, 95% CI 0.74 to 1.82, p = 0.51, I^2^ = 0%
		Test for subgroup difference: I^2^ = 0%
1-year OS rate	3	HR 1.15, 95% CI 0.75 to 1.75, p = 0.52, I^2^ = 0%
1-year DFS rate	3	HR 1.07, 95% CI 0.76 to 1.50, p = 0.70, I^2^ = 0%
3-year survival rate	7	HR 1.02, 95% CI 0.87 to 1.20, p = 0.78, I^2^ = 0%
Partial gastrectomy	5	HR 1.05, 95% CI 0.87 to 1.27, p = 0.61, I^2^ = 0%
Total gastrectomy	1	HR 0.98, 95% CI 0.59 to 1.62, p = 0.94
Partial/total gastrectomy	1	HR 0.95, 95% CI 0.66 to 1.36, p = 0.78
		Test for subgroup difference: I^2^ = 0%
<Stage III	4	HR 1.04, 95% CI 0.85 to 1.28, p = 0.70, I^2^ = 0%
≥Stage III	4	HR 1.00, 95% CI 0.81 to 1.23, p = 0.97, I^2^ = 0%
		Test for subgroup difference: I^2^ = 0%
3-year OS rate	5	HR 1.02, 95% CI 0.84 to 1.25, p = 0.82, I^2^ = 0%
3-year DFS rate	5	HR 1.05, 95% CI 0.91 to 1.21, p = 0.51, I^2^ = 0%
5-year survival rate	4	HR 1.10, 95% CI 0.90 to 1.36, p = 0.35, I^2^ = 0%
Partial gastrectomy	2	HR 1.12, 95% CI 0.89 to 1.41, p = 0.33, I^2^ = 0%
Total gastrectomy	1	HR 0.89, 95% CI 0.21 to 3.84, p = 0.88
Partial/total gastrectomy	1	HR 1.05, 95% CI 0.64 to 1.72, p = 0.85
		Test for subgroup difference: I^2^ = 0%
<Stage III	2	HR 1.30, 95% CI 0.90 to 1.87, p = 0.16, I^2^ = 0%
≥Stage III	2	HR 1.07, 95% CI 0.87 to 1.31, p = 0.54, I^2^ = 0%
		Test for subgroup difference: I^2^ = 0%
5-year OS rate	4	HR 1.10, 95% CI 0.90 to 1.36, p = 0.35, I^2^ = 0%
5-year DFS rate	3	HR 1.05, 95% CI 0.84 to 1.22, p = 0.86, I^2^ = 0%

N, number of studies; ICU, intensive care unit; OR, odds ratio; CI, confidence interval; HR, hazard ratio; MD, mean difference; OS, overall survival; DFS, disease-free survival; LN, lymph node.

Data on the short-term mortality were reported in ten trials, and in five trials the mortality was 0% for both groups. A meta-analysis of the remaining five trials revealed no differences in short-term mortality between the groups (OR 0.72, 95% CI 0.34 to 1.54, p = 0.39, I^2^ = 0%; **Table 2**, **Figure 3C**).

**Figure 4:**
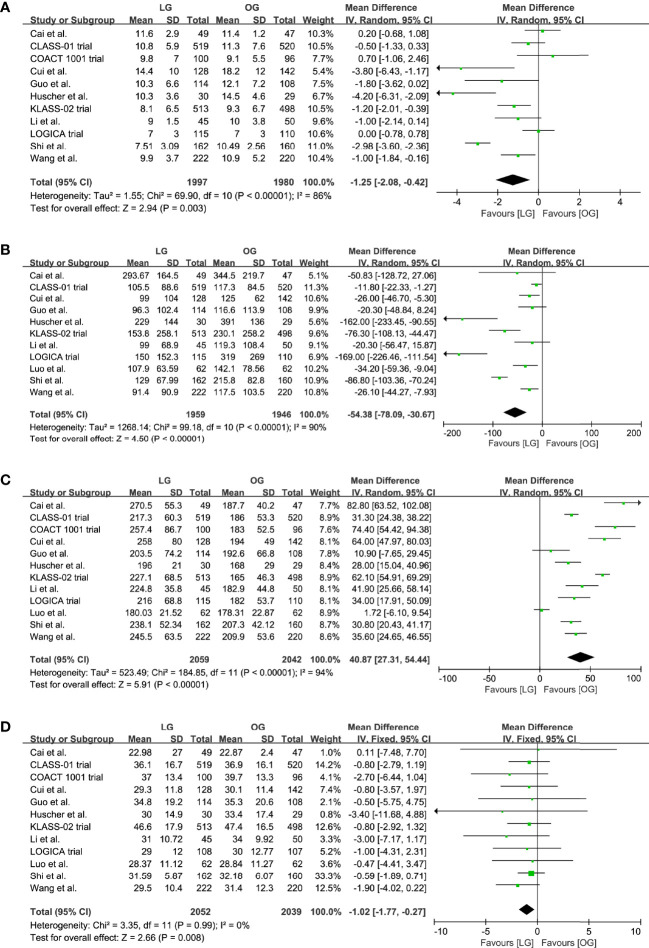
Pooled estimates of **(A)** length of hospital stay, **(B)** blood loss, **(C)** surgical time, **(D)** number of retrieved lymph nodes.

Eleven trials reported the data on length of hospital stay and blood loss; our results indicated that the LG was associated with a shorter length of hospital stay (MD -1.25, 95% CI -2.08 to -0.42, p = 0.003, I^2^ = 86%; **Table 2**, **Figure 4A**) and reduced blood loss (MD -54.38, 95% CI -78.09 to -30.67, p < 0.00001, I^2^ = 90%; **Table 2**, **Figure 4B**).

All trials reported a longer surgical time of the LG group, and our meta-analysis further confirmed this effect (MD 40.87, 95% CI 27.31 to 54.44, p < 0.00001, I^2^ = 94%; **Table 2**, **Figure 4C**). However, these results should be interpreted prudently because of the significant heterogeneity.

In addition, the LG group had a lower number of retrieved lymph nodes when compared with the OG group (MD -1.02, 95% CI -1.77 to -0.27, p = 0.008, I^2^ = 0%; **Table 2**, **Figure 4D**).

### Long-Term Outcomes

**Figure 5:**
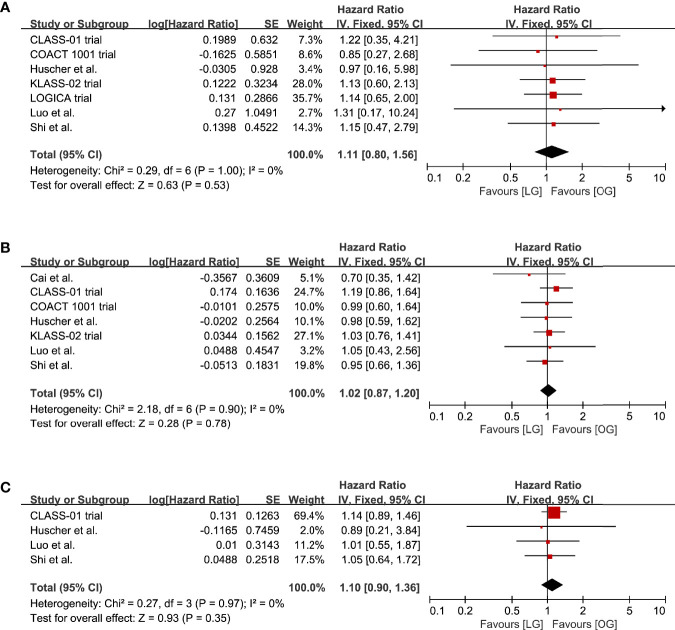
Pooled estimates of survival rates at **(A)** 1-year, **(B)** 3-year, **(C)** 5-year.

The survival rates at 1, 3, and 5 years were reported in eight studies. The LG group had survival rates of 91.6%, 64.7%, and 32.9% at 1, 3, and 5 years. The survival rates in the OG group were 89.0%, 59.1%, and 31.4%, respectively. The meta-analysis indicated that there was no significant difference in the survival rates at 1, 3, and 5 years between the LG and OG groups (1-year: HR 1.11, 95% CI 0.80 to 1.56, p = 0.53, I^2^ = 0%; 3-year: HR 1.02, 95% CI 0.87 to 1.20, p = 0.78, I^2^ = 0%; 5-year: HR 1.10, 95% CI 0.90 to 1.36, p = 0.35, I^2^ = 0%; **Table 2**, **Figure 5**).

Furthermore, we stratified survival data by OS or DFS rate; the pooled results showed no significant difference in the OS or DFS rate at 1, 3, and 5 years between the LG and OG groups, respectively (**Supplementary Material 4**).

### Subgroup and Sensitivity Analyses

Predefined subgroup analyses stratified by the extent of resection (partial versus total gastrectomy) and tumor stage (clinical stage II versus stage III) were performed to explore the potential discrepant treatment effect of different subgroups (**Table 2**, **Supplementary Material 5**). In addition, based on the Clavien–Dindo classification (grades I to II as minor complications, grades III to V as major complications) (34), we divided the data of postoperative complications into minor and major complications.

The extent of resection had no effect on the overall postoperative complications, anastomotic leakage, short-term mortality, and long-term outcomes. Similarly, there was no significant difference in minor and major complications between the LG and OG groups. The beneficial effect of LG in reducing the length of hospital stay and blood loss was more significant after total gastrectomy than partial gastrectomy. Moreover, compared with total gastrectomy, patients receiving partial gastrectomy by a laparoscopic route was associated with more significantly longer surgical time and lower number of retrieved lymph nodes than open surgery.

Regarding tumor stage, five trials (11–13, 21, 25) provided the long-term survival data of clinical stage II and III gastric cancer, respectively. The results of subgroup analyses showed no significant difference for survival rates at 1, 3, and 5 years between LG and OG groups in both clinical stage II and III gastric cancer population.

In the sensitivity analysis, the LG was relevant to the obvious decrease in postoperative complications (OR 0.77, 95% CI 0.63 to 0.95) after omitting the CLASS-01 trial, indicating the poor robustness. Furthermore, other short- and long-term outcomes showed no significant differences with primary results (**Supplementary Material 6**).

## Discussion

There is growing high-quality RCTs supporting the feasibility of LG for AGC, and its safety is confirmed in our study. In this up-to-date meta-analysis, we reviewed 12 RCTs with 4,101 patients to compare the short- and long-term outcomes of LG versus OG. The result shows that the LG significantly reduces the length of hospital stay and blood loss, whereas the number of retrieved lymph nodes was lower and surgical time was longer in the LG group. Furthermore, there were no differences between LG and OG groups in terms of postoperative complications, short-term mortality, and survival rate at 1, 3, and 5 years. The finding provides further evidence for the safety and efficacy of LG for the treatment of ACG.

To the best of our knowledge, this study is not the first meta-analysis of RCTs to compare the LG with OG for the treatment of gastric cancer. Recently, Vasavada and Patel (35) performed an updated meta-analysis of 11 RCTs (6 RCTs for EGC and 5 RCTs for AGC); the results demonstrated that the LG was associated with lesser wound-related complications without decreasing the length of hospital stay. However, for the patients with AGC, most trials analyzed in previous meta-analyses were non-randomized, which may increase the risk of potential selection and publication bias. Therefore, the current meta-analysis provides the most comprehensive and accurate analysis, since it sums up the up-to-date and high-quality RCTs in terms of comparing LG to OG for patients with AGC. Also, compared with previous meta-analyses, more RCTs with a long-term follow-up were included in it. In general, our results are in compliance with previous meta-analyses (36–40), showing that when compared to the open approach, the LG provides improved short-term outcomes and similar long-term outcomes in patients with AGC.

When choosing the clinical outcomes of our study, we compared the LG with OG on different levels in terms of safety (postoperative morbidity), difficulty (operative time, blood loss, number of retrieved lymph nodes), efficiency (length of hospital stay), and its long-term oncologic results (OS and DFS rates). The results of our meta-analysis indicate that the short-term outcomes consisting of blood loss and length of hospital stay are in favor of a laparoscopic approach, especially for total gastrectomy. Since the advanced laparoscopic approach provides a magnified surgical view while minimizing the length of the incision, a more delicate surgical manipulation of the organs, vessels, and nerves could be achieved during operation (23). In addition, the reduction of hospital stay may be a combined result of fewer blood loss during operation, faster postsurgical recovery of bowel function, and lighter postoperative pain (36).

The overall postoperative complications, including minor and major complications, were similar between the two surgical procedures in our study. However, a recent meta-analysis of data from 6 RCTs and 18 non-randomized trials found that LG was associated with a lower postoperative complication rate, with a significantly lower incidence of medical and minor surgical complications (36). The difference may result from several newly published RCTs, especially the CLASS-01 (16) and LOGICA trials (13). In the sensitivity analysis, we found a significant difference for the postoperative complications in the LG group after omitting the CLASS-01 trial (41). Considering the high risk of imprecision bias, more evidence about the effect of LG on postoperative complications is compellingly needed in the future. Furthermore, anastomotic leakage, the major postoperative complication of gastric surgery, was comparable between two groups. Notably, although the observed difference was meaningless from the statistical perspective, anastomotic leakage seemed to have a higher possibility to occur after laparoscopic surgery. It highlights that this potential risk should be put more emphasis on. There are studies that propose that the application of mini-laparotomy for extracorporeal anastomosis in laparoscopic surgery for AGC may result in increased surgical difficulty, which may increase the likelihood of anastomotic drawbacks on the other hand (41).

Based on the updated studies, LG requires a longer surgical time, which is in line with the results of all included trials. Compared with open approaches, laparoscopic techniques are more complex and less flexible. Frequently cleaning cameras and changing instruments during operation can also extend the surgical time (42, 43). In addition, it is a challenge to perform the dissection of enlarged or suprapancreatic lymph nodes through the laparoscopic approach, as it is difficult to follow the no-touch principle for laparoscopic lymphadenectomy at a deep lymph node station. Moreover, due to the restriction of the visibility and the narrowness of the peritoneal cavity, total omentectomy in LG is also hard to achieve compared with that in OG.

Although the result of our meta-analysis indicates that the number of retrieved lymph nodes was lower in the LG group, the mean number of retrieved lymph nodes in the LG group was 32.45 (95% CI 29.01 to 35.89). Based on the American Joint Committee on Cancer, an adequate dissection should include at least 15 lymph nodes for patients with gastric cancer to ensure accurate and robust N staging (44). A recent study demonstrated that an examined lymph node threshold of more than 30 was shown to be beneficial of survival for patients with gastric cancer and should be considered a clinical benchmark for practice (45). It is in accordance with the most crucial results of our study in terms of the long-term outcomes. The 1-, 3, and 5-year survival rates were similar among the two groups. When the surgical margins fulfilled the R0 resection criteria and adequate lymph node dissection could be achieved, long-term survival largely depended on the intrinsic biological characteristic of the cancer rather than the surgical approach (46, 47).

Furthermore, the learning curve was proved to have significant effects on most of the important surgical and short-term recovery outcome parameters (48). Yoo et al. performed a prospective study to estimate the learning curve of LG for EGC, indicating that surgeons who performed at least 50 cases of LG could achieve lower postoperative complications, more resected lymph nodes, shorter surgical time, and postoperative hospital stay (49). Thus, the LG should be restricted to specialist centers where adequate training and supervision could be provided during the learning curve.

However, some limitations of our meta-analysis must be acknowledged. First of all, the sample size of some included trials was relatively small, which may decrease the credibility of the results in our study or lead to small study effect bias (50). Secondly, our study did not consider the differences and potential impact of surgeons’ experience, perioperative care protocols, and surgical technique between studies, despite their application having been shown to be beneficial in many studies (51–53). Thirdly, most of all included trials were conducted in East Asia (eight in China, two in Korea), except one in Italy and one in Netherlands. Therefore, the generalizability of the findings to Western countries may be limited. Moreover, since the Western population has a comparatively low incidence of gastric cancer, higher body mass index, and more comorbidities (54), the results may not necessarily apply in the Western population.

Last but not least, there was significant heterogeneity in some pooled estimates, which might be explained by differences in sample sizes, surgeons’ experience, perioperative care protocols, surgical technique, pre- and postoperative chemotherapy, and other factors. Variations in sample size among studies were large, and some studies enrolled patients during a wide study interval, which may have introduced biases due to a progression in mastering the surgical skills and improvements in surgical instruments.

## Conclusion

Our findings, which are contingent on rigorous meta-analyses of high-quality RCTs, suggest that LG offers improved short-term outcomes including shorter hospital stays and fewer blood loss, with comparable postoperative complications, short-term mortality, and long-term survival rates when compared to the open approach. However, considering the significant heterogeneity, more RCTs are needed to further evaluate the clinical outcomes of LG versus OG for patients with AGC. Furthermore, this updated meta-analysis could be the basis of future meta-analyses, as the inclusion criteria, statistical analysis, and short- and long-term outcomes were clearly defined and meticulously analyzed.

## Data Availability Statement

The original contributions presented in the study are included in the article/**Supplementary Material**. Further inquiries can be directed to the corresponding author.

## Author Contributions

JJ and SW conceived the idea, performed the analysis, and drafted the initial draft writing of this paper. GY, JW, and XX contributed to the collection and interpretation of data. KZ helped to frame the idea of the study and provided technical support. SW contributed to the revision of this paper and the final approval of the version to be published. All authors contributed to the article and approved the submitted version.

## Conflict of Interest

The authors declare that the research was conducted in the absence of any commercial or financial relationships that could be construed as a potential conflict of interest.

## Publisher’s Note

All claims expressed in this article are solely those of the authors and do not necessarily represent those of their affiliated organizations, or those of the publisher, the editors and the reviewers. Any product that may be evaluated in this article, or claim that may be made by its manufacturer, is not guaranteed or endorsed by the publisher.
