# Supplementary material for: The Comparison of Short- and Long-Term Outcomes for Laparoscopic Versus Open Gastrectomy for Patients With Advanced Gastric Cancer: A Meta-Analysis of Randomized Controlled Trials
Source: Front Oncol. 2022 Apr 5;12:844803. doi: 10.3389/fonc.2022.844803 (PMC9016843; doi:10.3389/fonc.2022.844803)
Supplement: Supplementary file 2 [file DataSheet_2.docx]

**Supplementary Material 2:** Searching strategies

Pubmed 239

#1 Stomach Neoplasms [MeSH] OR Gastric Cancer [Title/Abstract] OR gastric carcinoma [Title/Abstract] OR stomach cancer [Title/Abstract]

#2 Laparoscopic [Title/Abstract] OR Laparoscopy [MeSH] OR Laparoscopy [Title/Abstract]

#3 randomized [Title/Abstract]

#4 open [Title/Abstract]

#1 AND #2 AND #3 AND #4

Embase 361

#1 'stomach cancer'/exp OR 'gastric cancer':ab,ti OR 'gastric carcinoma':ab,ti OR 'stomach cancer':ab,ti

#2 ‘Laparoscopic’:ab,ti OR ‘Laparoscopy’:ab,ti OR ‘Laparoscopy’/exp

#3 (randomized control trial):ab,ti OR ‘random*’:ab,ti

#1 AND #2 AND #3

Scopus 383

#1 TITLE-ABS-KEY (stomach cancer) OR TITLE-ABS-KEY (gastric cancer) OR TITLE-ABS-KEY (gastric carcinoma)

#2 TITLE-ABS-KEY (Laparoscopic) OR TITLE-ABS-KEY (Laparoscopy)

#3 TITLE-ABS-KEY (random*) OR TITLE-ABS-KEY (randomized control trial)

#4 TITLE-ABS-KEY (open)

#1 AND #2 AND #3 AND #4

Cochrane Library 584

#1 ‘stomach cancer’:ti,ab,kw OR ‘gastric cancer’: ti,ab,kw OR ‘gastric carcinoma’: ti,ab,kw OR ‘stomach cancer’: ti,ab,kw

#2 (Laparoscopic): ti,ab,kw OR ‘Laparoscopy’: ti,ab,kw

#3 ‘random*’: ti,ab,kw OR (randomized control trial): ti,ab,kw

#4 open: ti,ab,kw

#1 AND #2 AND #3 AND #4
