# Supplementary material for: The Comparison of Short- and Long-Term Outcomes for Laparoscopic Versus Open Gastrectomy for Patients With Advanced Gastric Cancer: A Meta-Analysis of Randomized Controlled Trials
Source: Front Oncol. 2022 Apr 5;12:844803. doi: 10.3389/fonc.2022.844803 (PMC9016843; doi:10.3389/fonc.2022.844803)
Supplement: Supplementary file 3 [file DataSheet_3.docx]

**Supplementary Material 3:** Publication bias assessment by funnel plot and Egger’s test


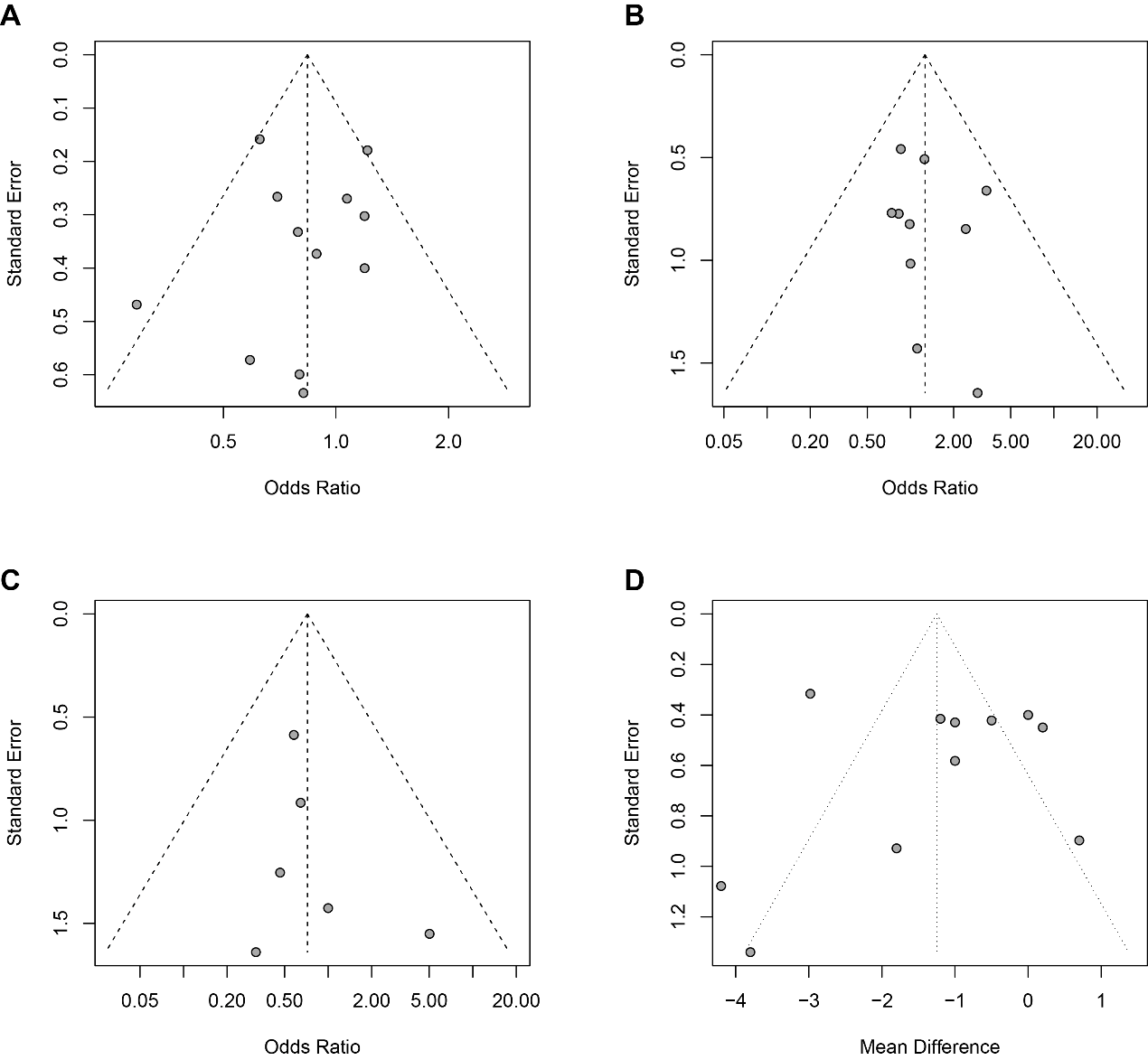


Figure 1: Funnel plot for (A) postoperative complications, Egger’s test P=0.7301; (B) anastomotic leakage, Egger’s test P=0.5519; (C) short-term mortality, Egger’s test P=0.4838; (D) length of hospital stay, Egger’s test P=0.9488


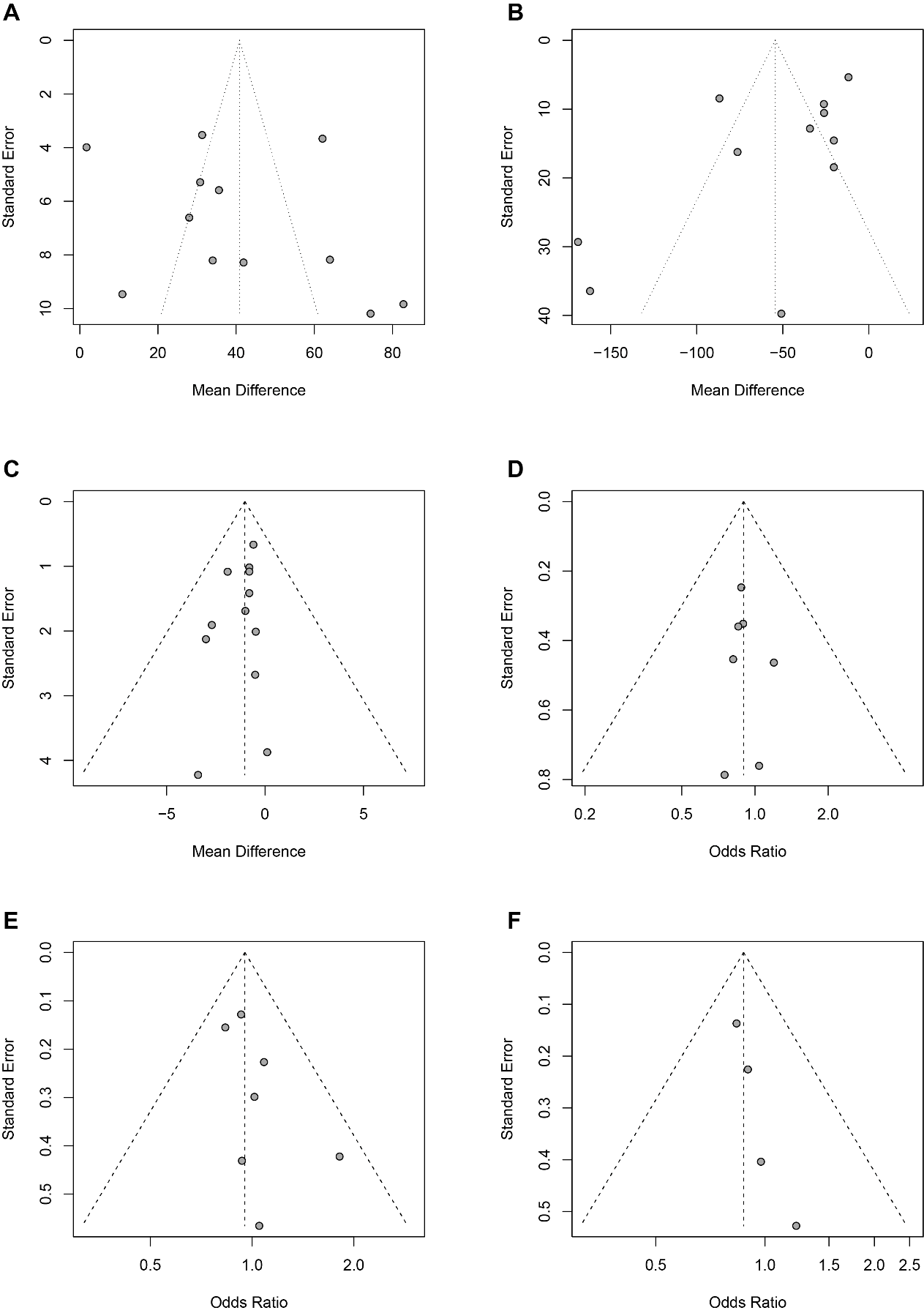


Figure 2: Funnel plot for (A) surgical time, Egger’s test P=0.4346; (B) blood loss, Egger’s test P=0.09634; (C) number of retrieved lymph nodes, Egger’s test P=0.1953; (D) 1-year survival rate, Egger’s test P=0.7275; (E) 3-year survival rate, Egger’s test P=0.1492; (F) 5-year survival rate, Egger’s test P=0.03133
