# Supplementary material for: The Comparison of Short- and Long-Term Outcomes for Laparoscopic Versus Open Gastrectomy for Patients With Advanced Gastric Cancer: A Meta-Analysis of Randomized Controlled Trials
Source: Front Oncol. 2022 Apr 5;12:844803. doi: 10.3389/fonc.2022.844803 (PMC9016843; doi:10.3389/fonc.2022.844803)
Supplement: Supplementary file 4 [file DataSheet_4.docx]

**Supplementary Material 4:** Overall survival rate versus disease-free survival rate at 1-, 3-, 5-year


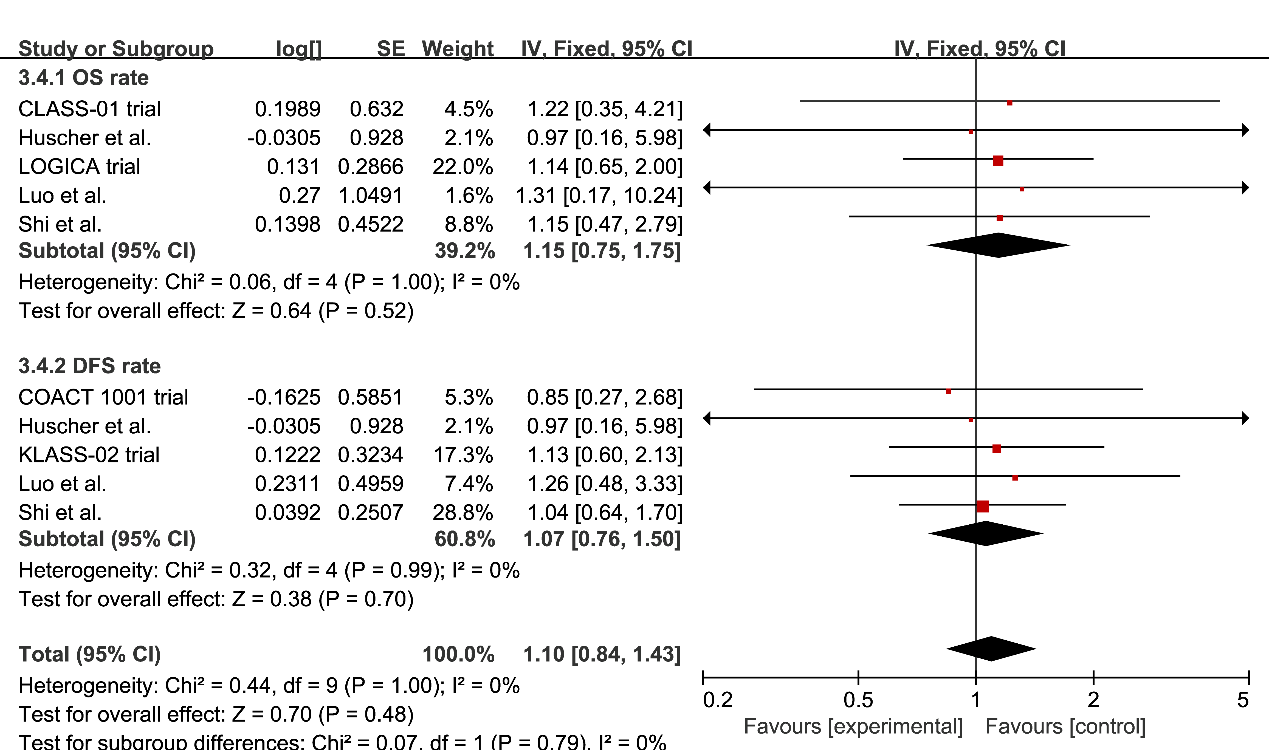


Figure 1: Subgroup analysis for 1-year survival rate, overall survival rate versus disease-free survival rate


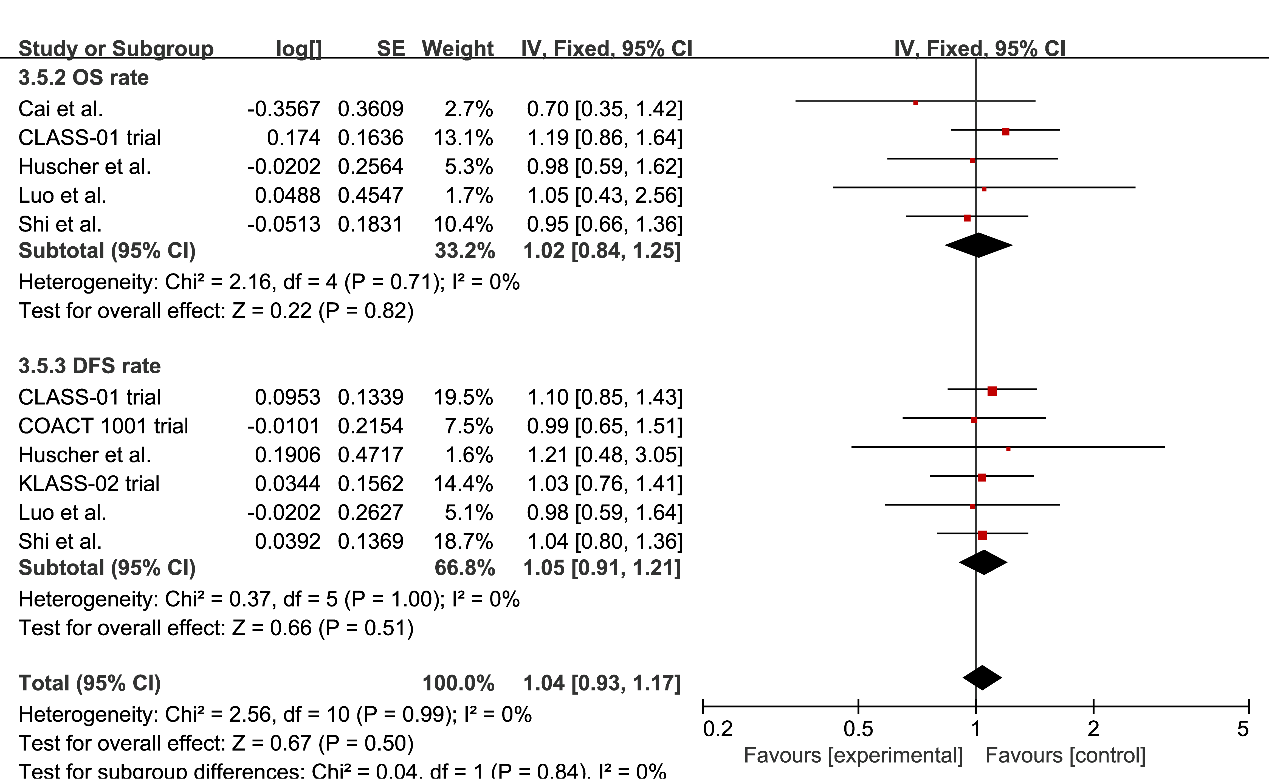


Figure 2: Subgroup analysis for 3-year survival rate, overall survival rate versus disease-free survival rate


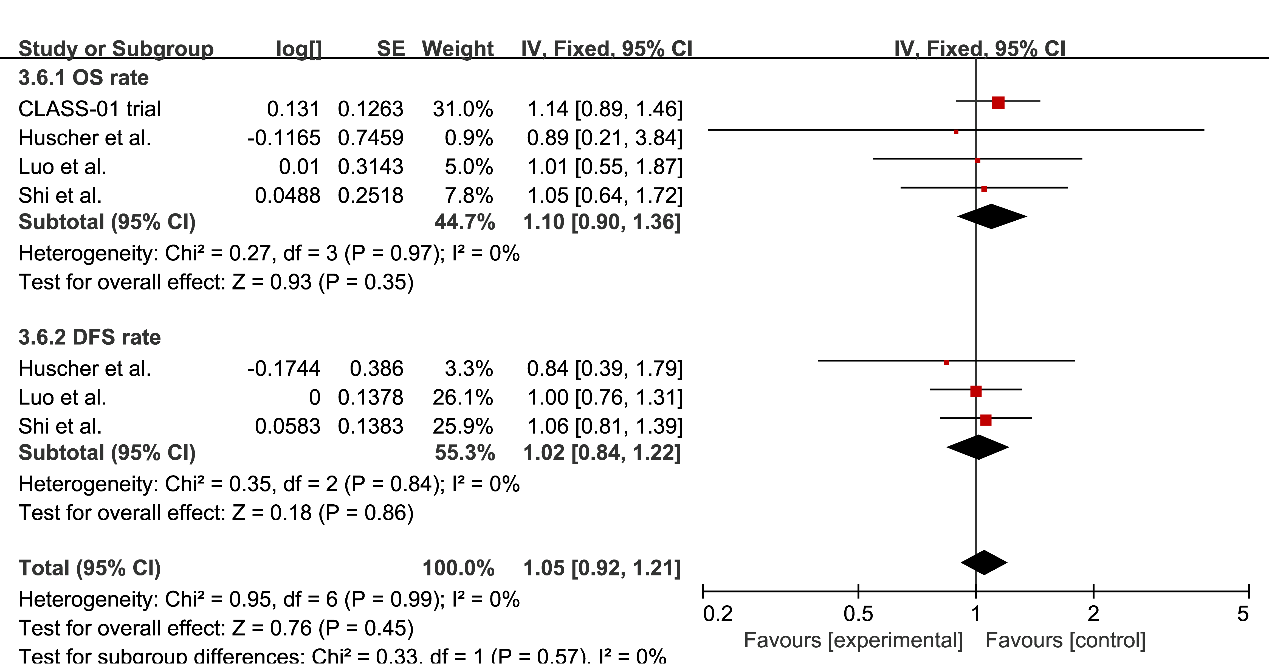


Figure 3: Subgroup analysis for 5-year survival rate, overall survival rate versus disease-free survival rate
