# Supplementary material for: The Comparison of Short- and Long-Term Outcomes for Laparoscopic Versus Open Gastrectomy for Patients With Advanced Gastric Cancer: A Meta-Analysis of Randomized Controlled Trials
Source: Front Oncol. 2022 Apr 5;12:844803. doi: 10.3389/fonc.2022.844803 (PMC9016843; doi:10.3389/fonc.2022.844803)
Supplement: Supplementary file 5 [file DataSheet_5.docx]

**Supplementary Material 5:** Subgroup analyses


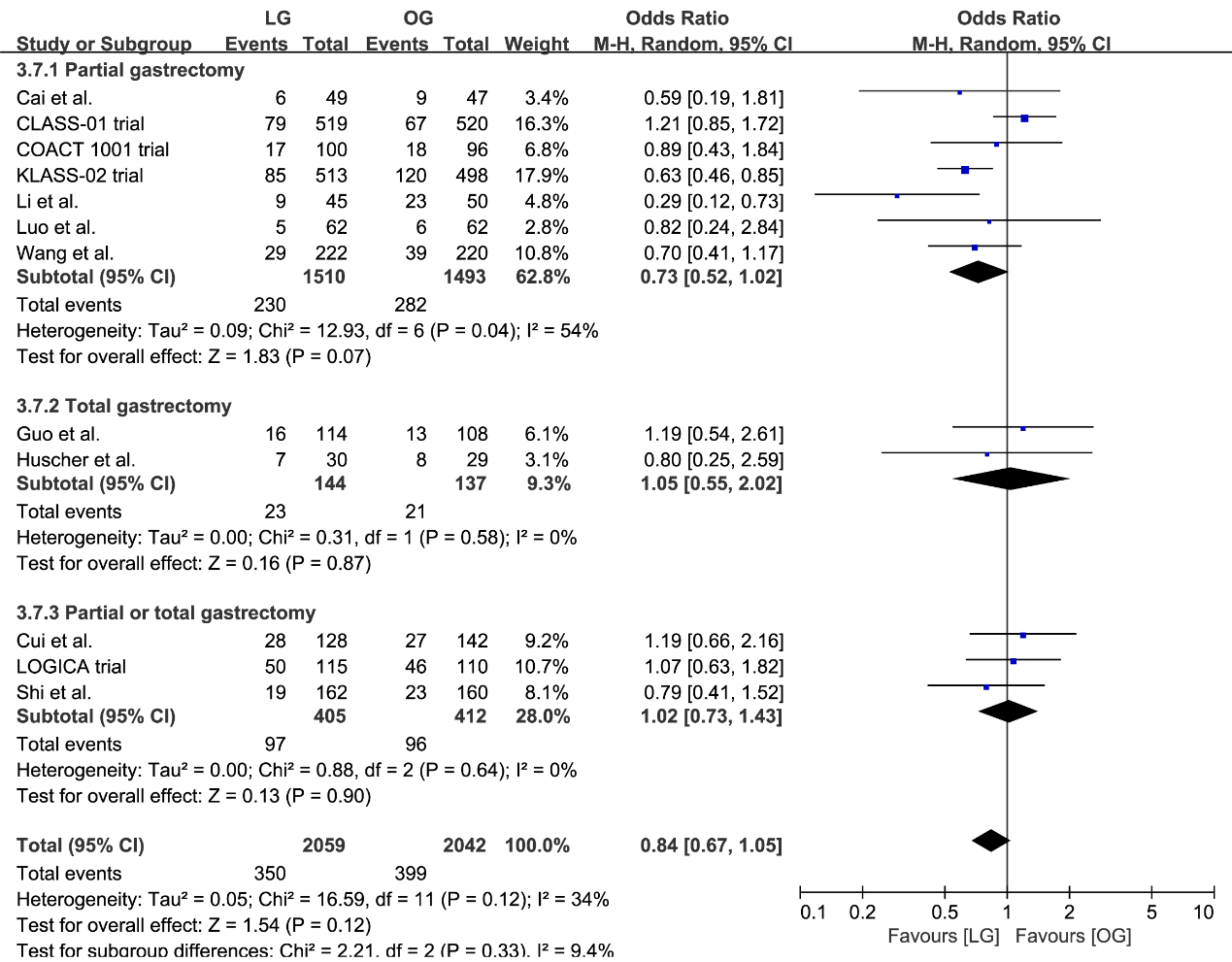


Figure 1: Subgroup analysis for postoperative complications, partial versus total versus partial or total gastrectomy


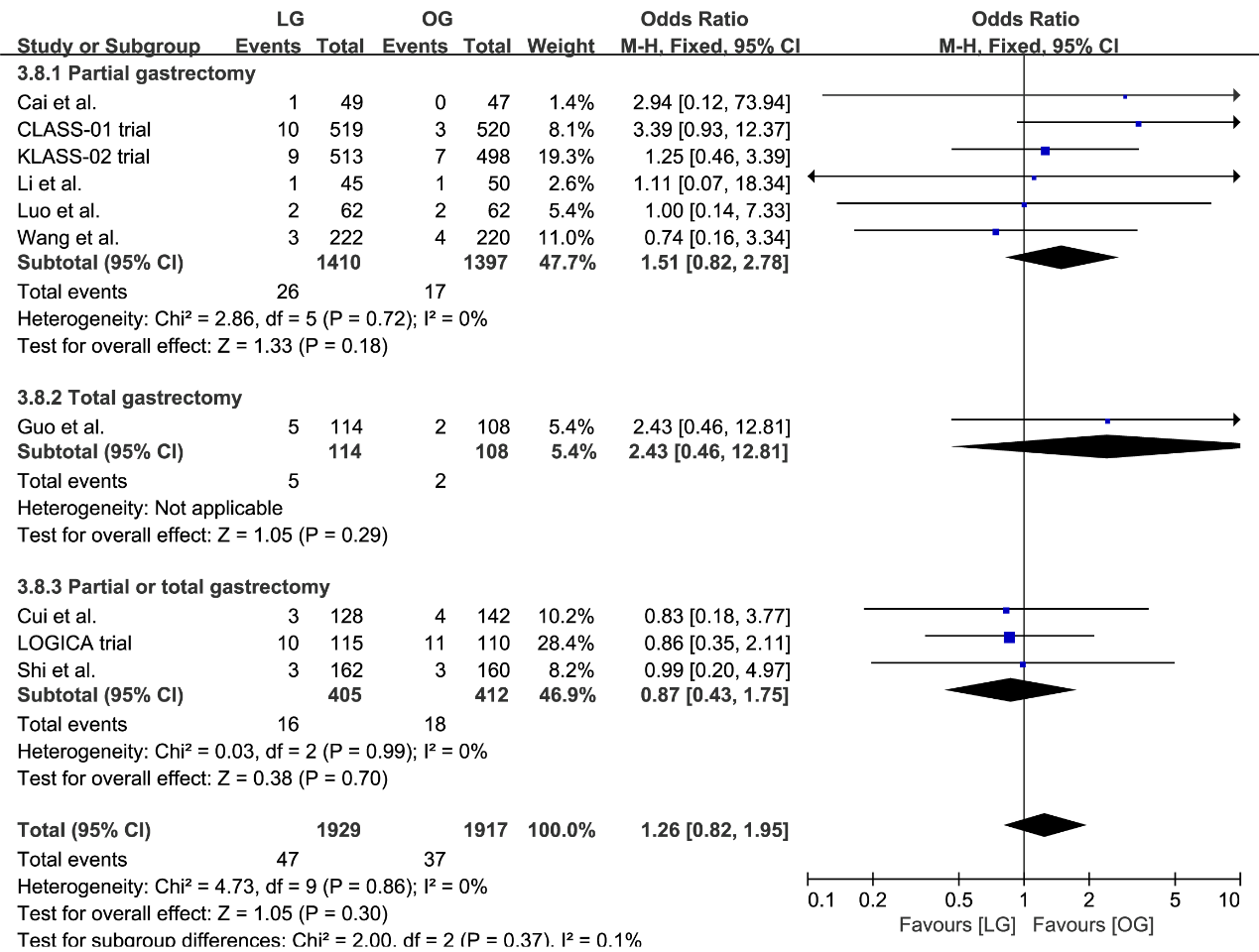


Figure 2: Subgroup analysis for anastomotic leakage, partial versus total versus partial or total gastrectomy


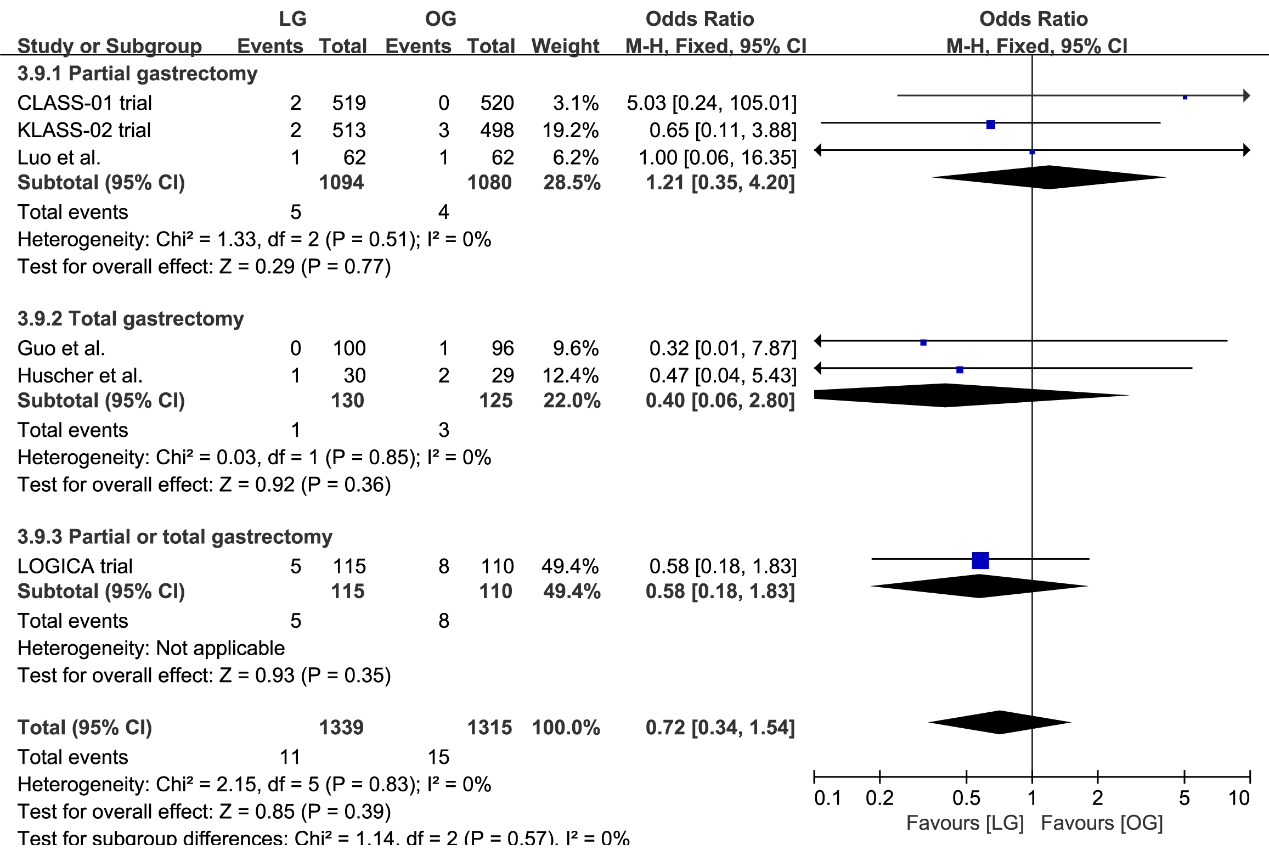


Figure 3: Subgroup analysis for short-term mortality, partial versus total versus partial or total gastrectomy


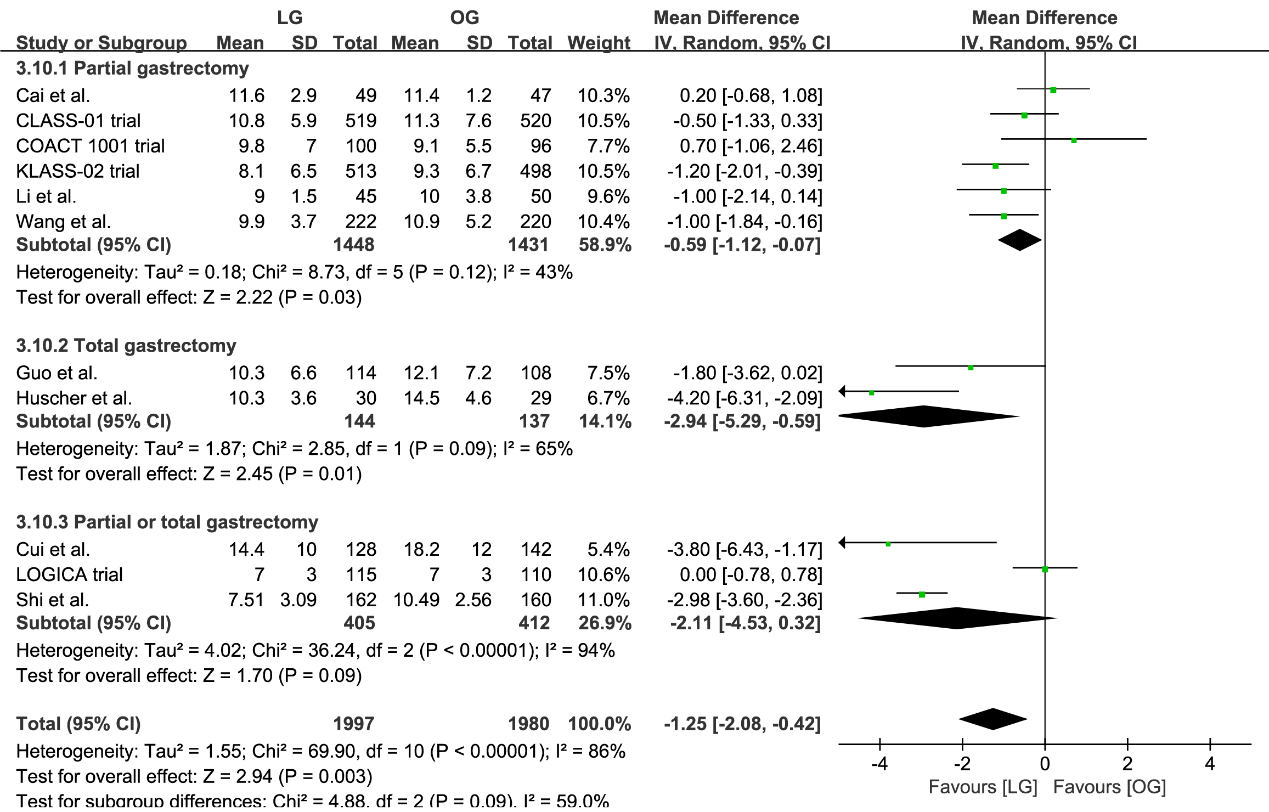
Figure 4: Subgroup analysis for length of hospital stay, partial versus total versus partial or total gastrectomy


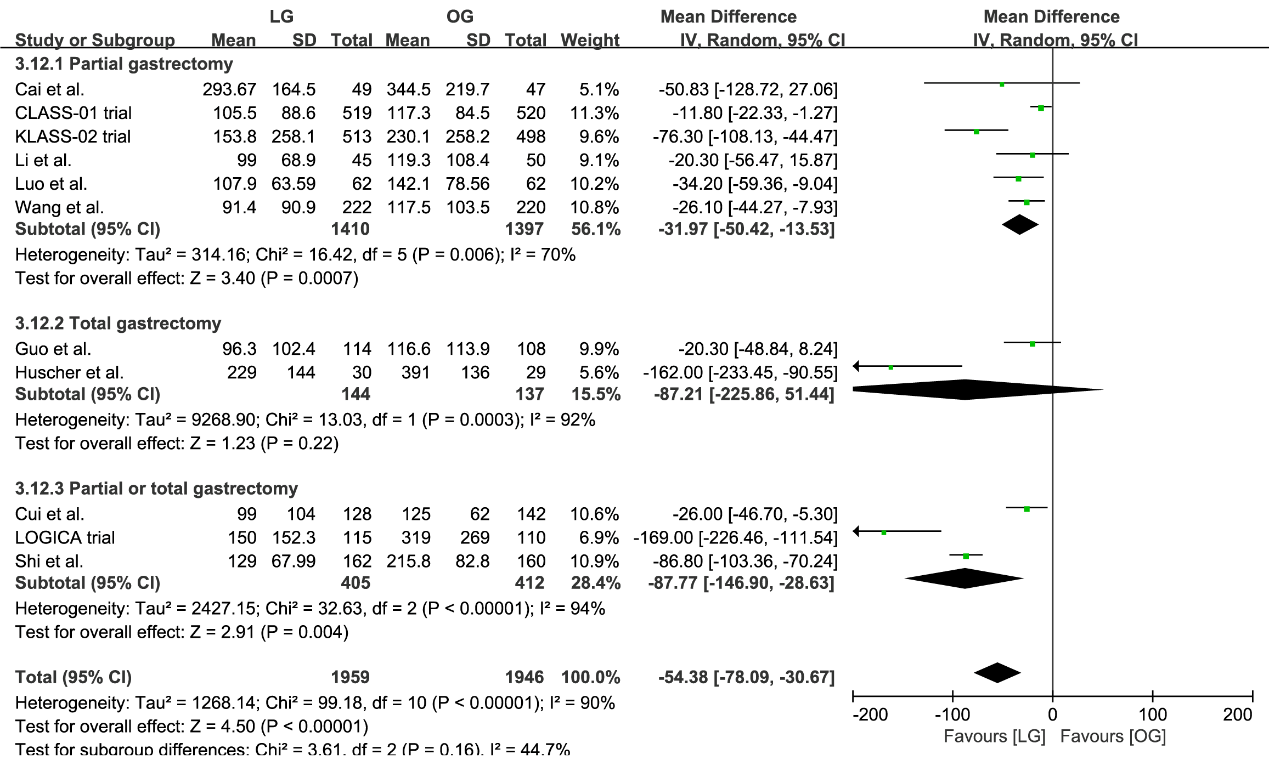


Figure 5: Subgroup analysis for blood loss, partial versus total versus partial or total gastrectomy


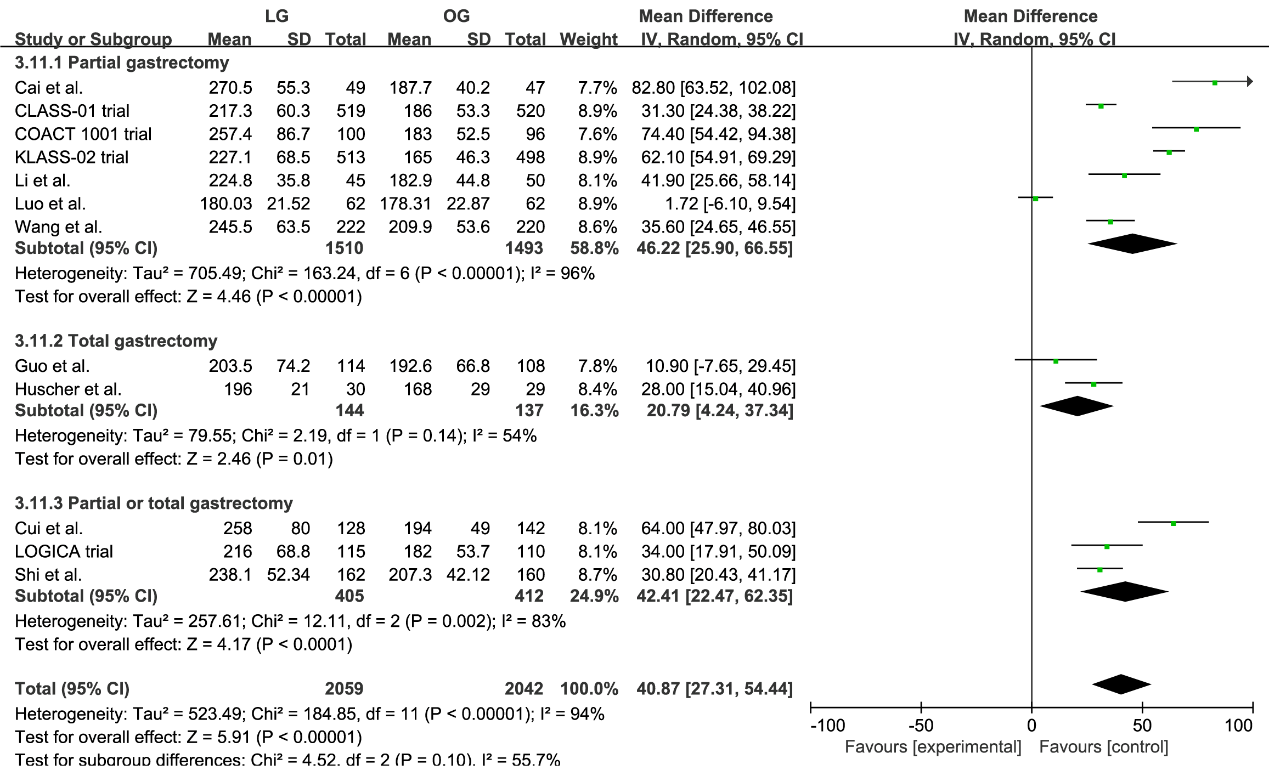
Figure 6: Subgroup analysis for surgical time, partial versus total versus partial or total gastrectomy


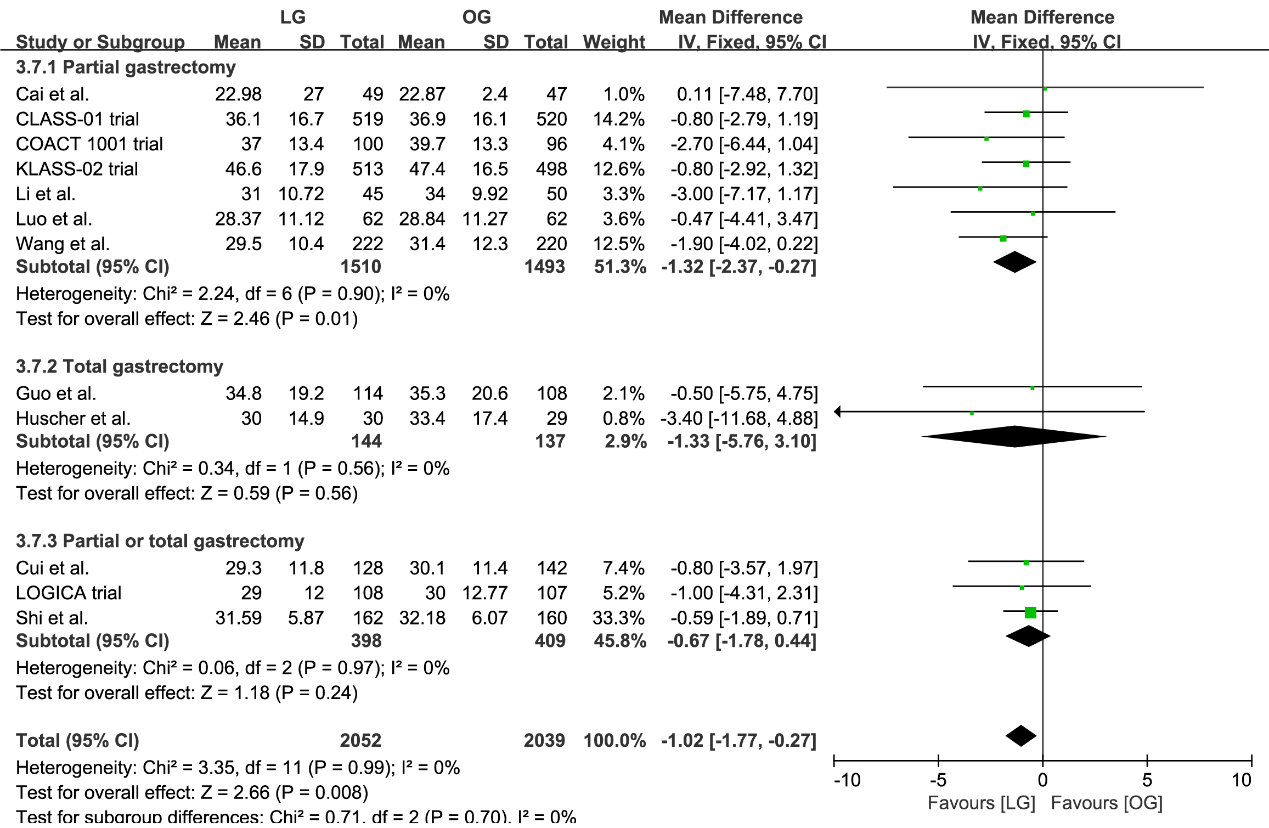


Figure 7: Subgroup analysis for number of retrieved lymph nodes, partial versus total versus partial or total gastrectomy


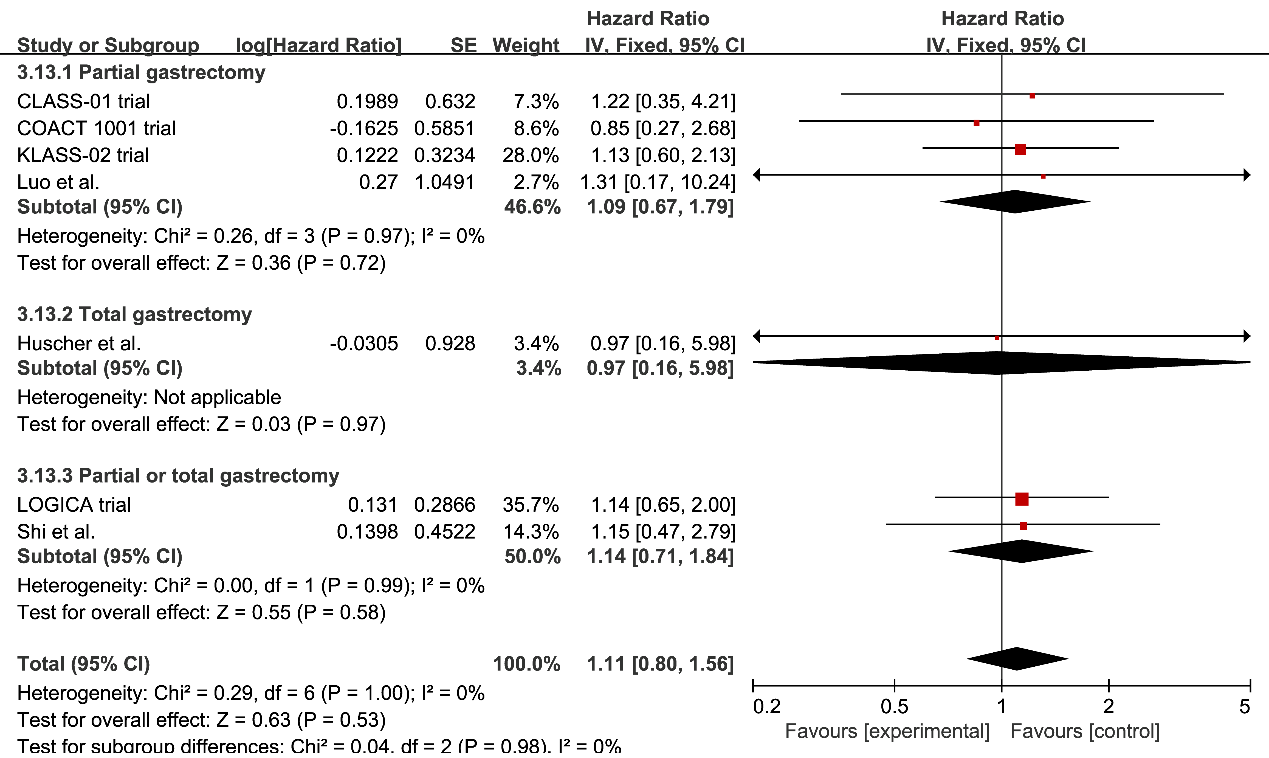
Figure 8: Subgroup analysis for 1-year survival rate, partial versus total versus partial or total gastrectomy


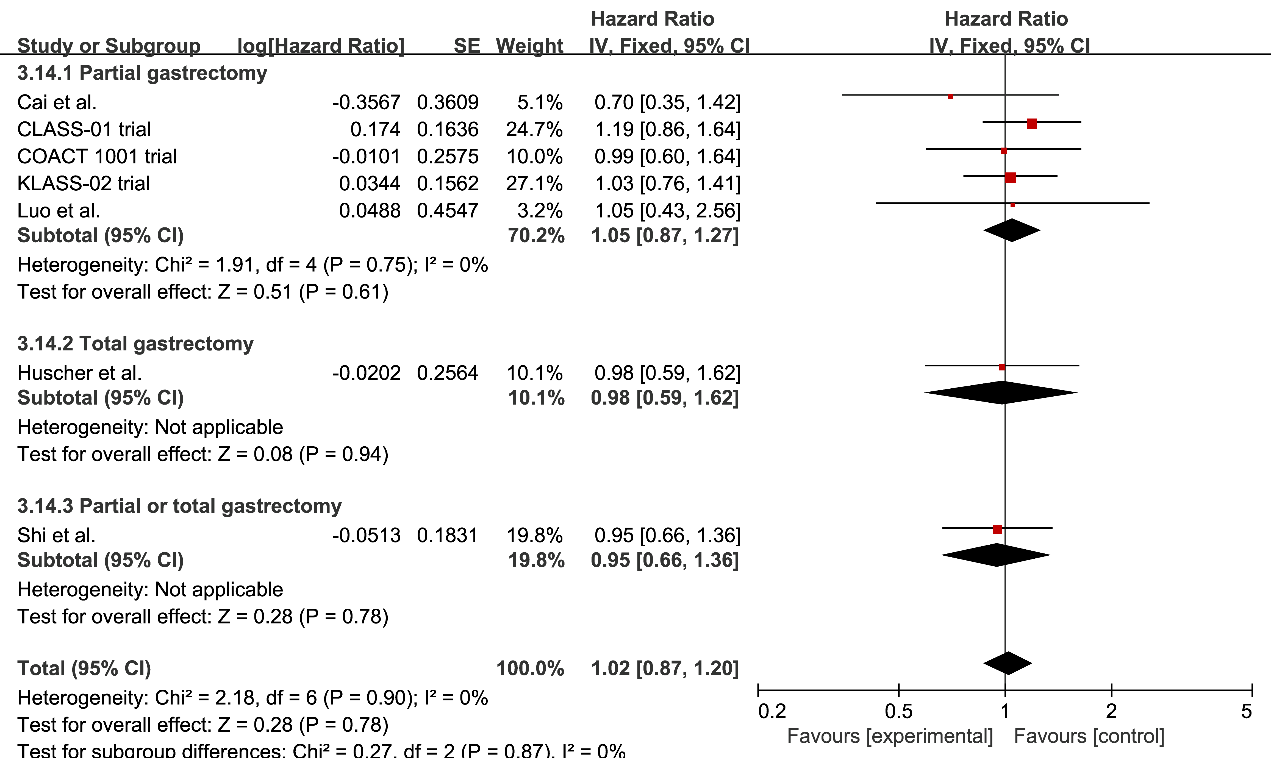


Figure 9: Subgroup analysis for 3-year survival rate, partial versus total versus partial or total gastrectomy


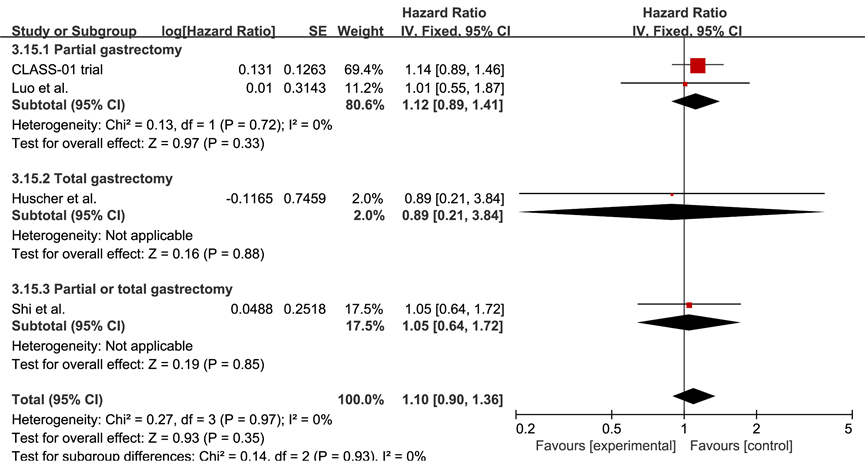


Figure 10: Subgroup analysis for 5-year survival rate, partial versus total versus partial or total gastrectomy


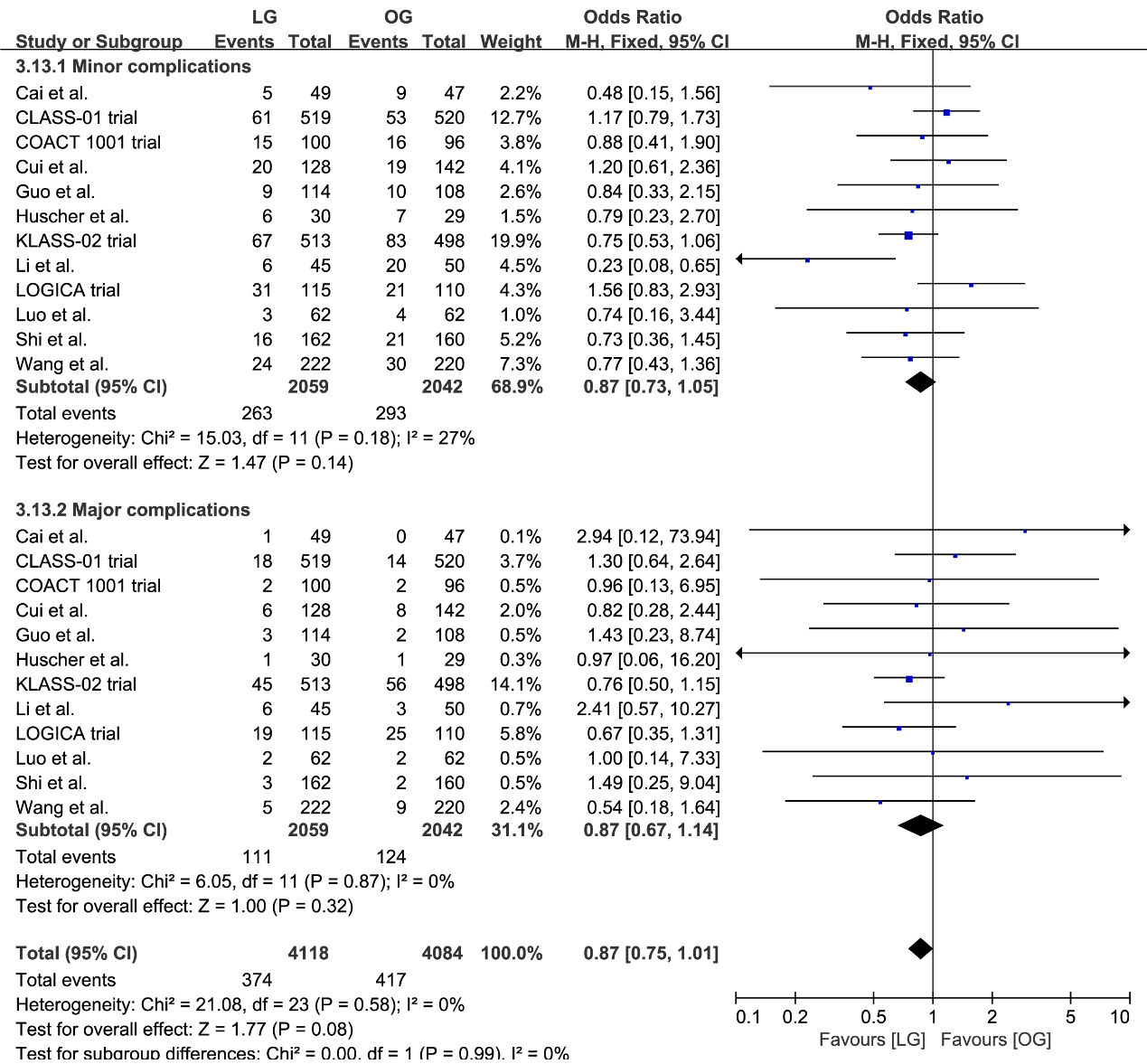


Figure 11: Subgroup analysis for postoperative complications, minor versus major complications


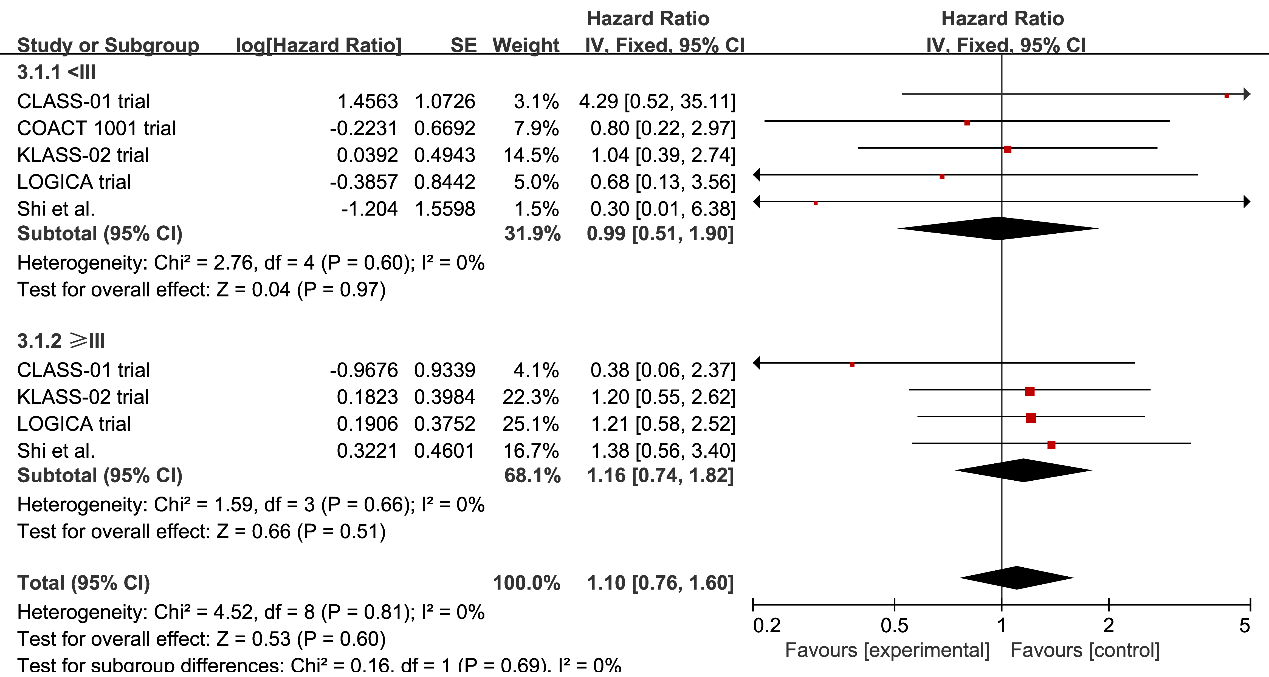


Figure 12: Subgroup analysis for 1-year survival rate, <III stage versus ≥III stage


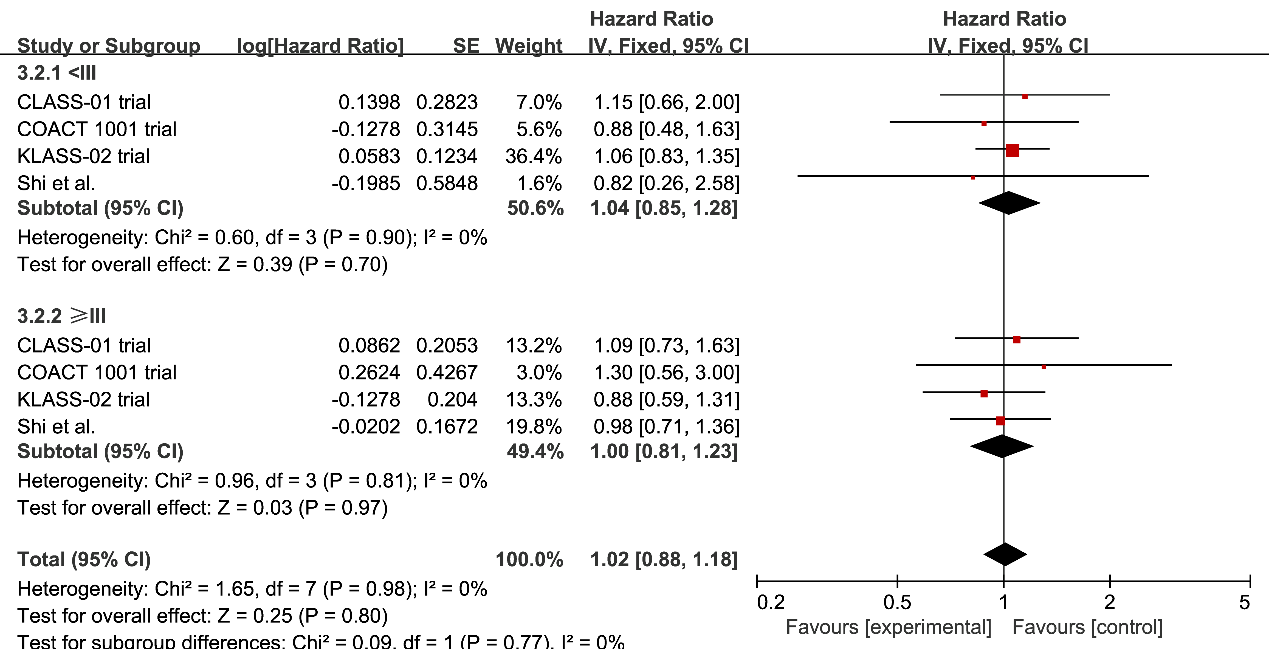


Figure 13: Subgroup analysis for 3-year survival rate, <III stage versus ≥III stage


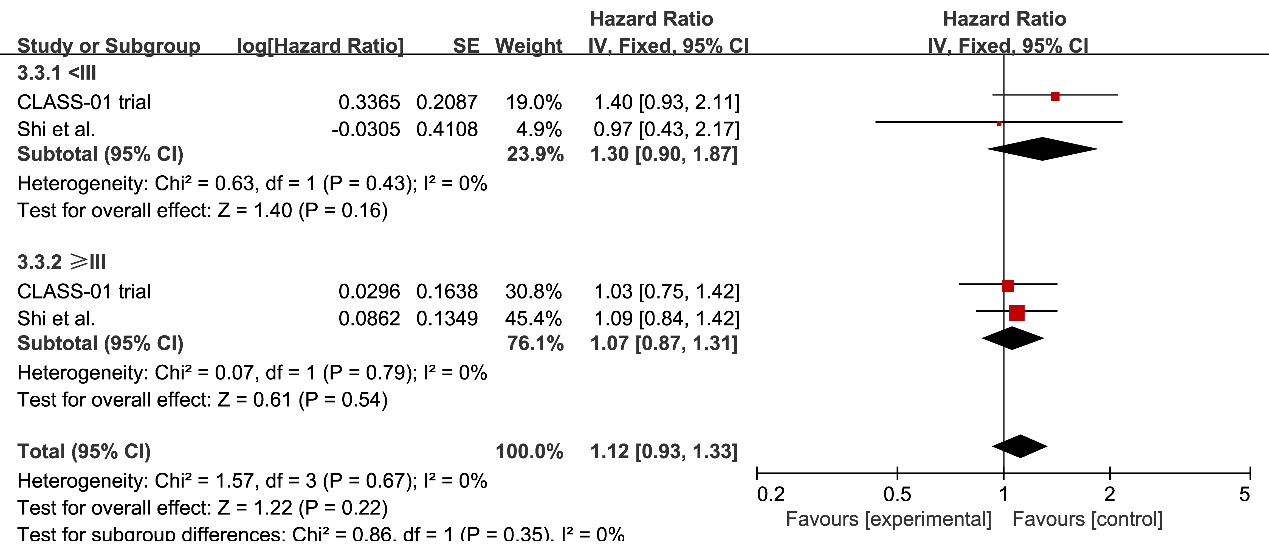


Figure 14: Subgroup analysis for 5-year survival rate, <III stage versus ≥III stage
