# Supplementary material for: The Comparison of Short- and Long-Term Outcomes for Laparoscopic Versus Open Gastrectomy for Patients With Advanced Gastric Cancer: A Meta-Analysis of Randomized Controlled Trials
Source: Front Oncol. 2022 Apr 5;12:844803. doi: 10.3389/fonc.2022.844803 (PMC9016843; doi:10.3389/fonc.2022.844803)
Supplement: Supplementary file 6 [file DataSheet_6.docx]

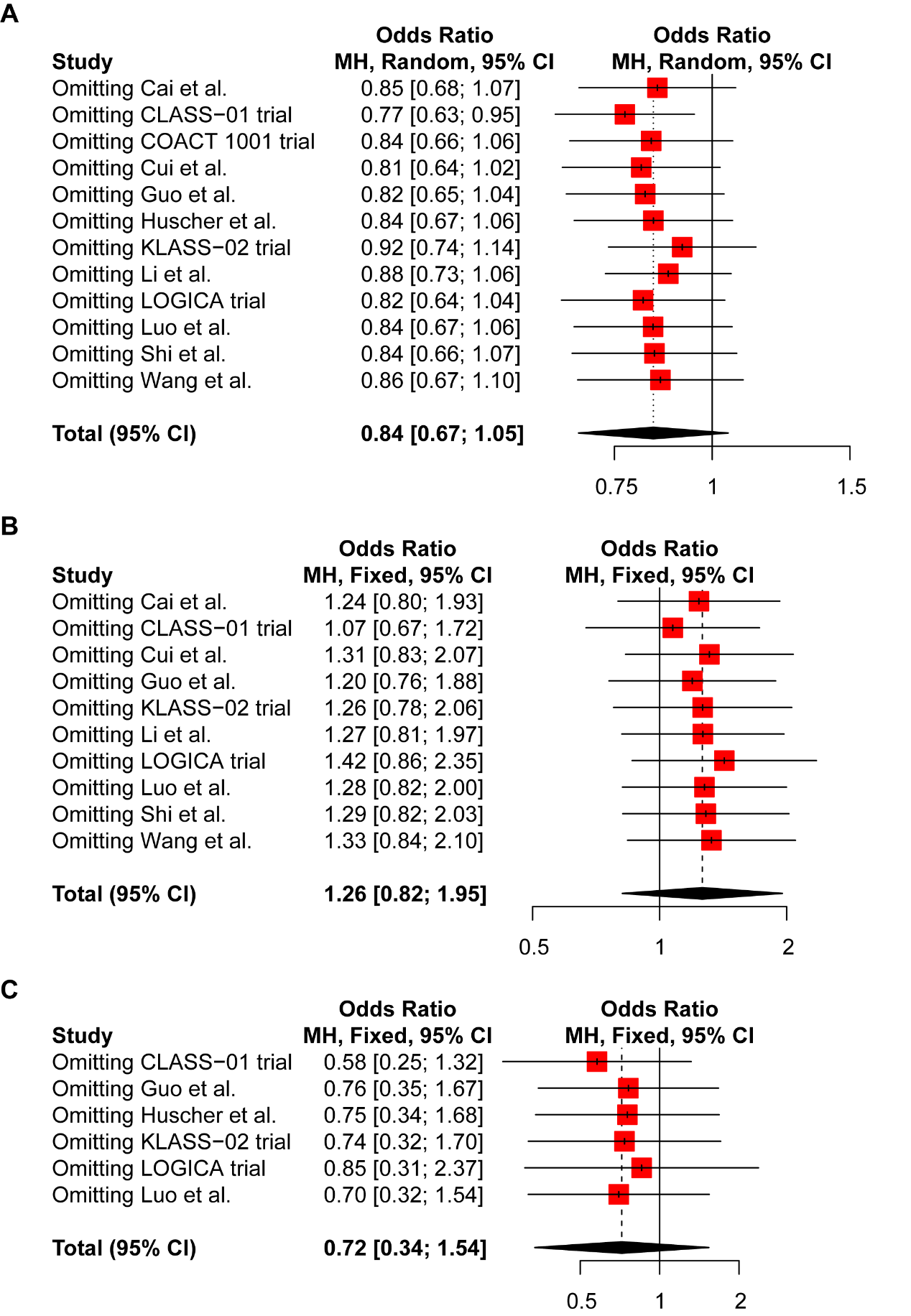
**Supplementary Material 6:** Sensitivity analyses

Figure 1: Sensitivity analysis for (a) postoperative complications; (b) anastomotic leakage; (c) short-term mortality


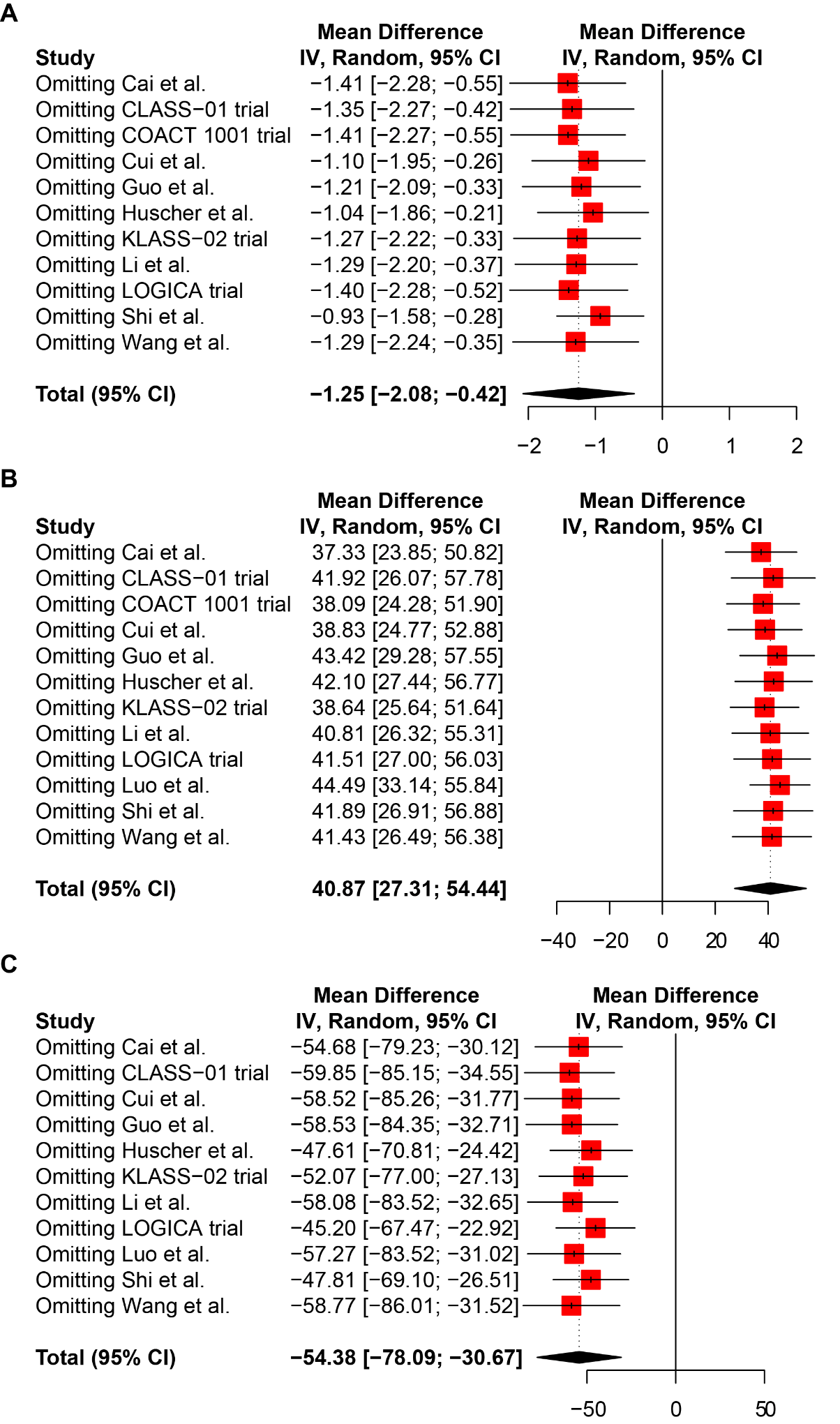


Figure 2: Sensitivity analysis for (a) length of hospital stay; (b) surgical time; (c) blood loss


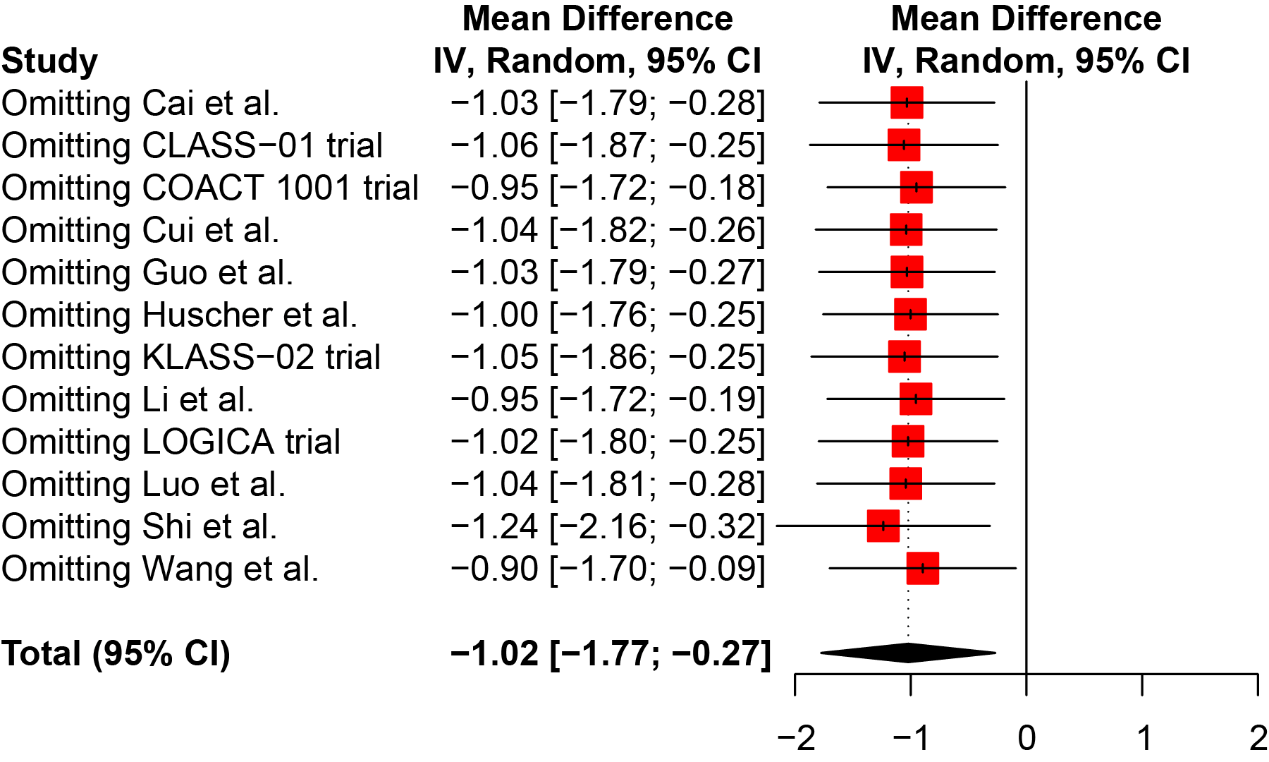


Figure 3: Sensitivity analysis for number of retrieved lymph nodes


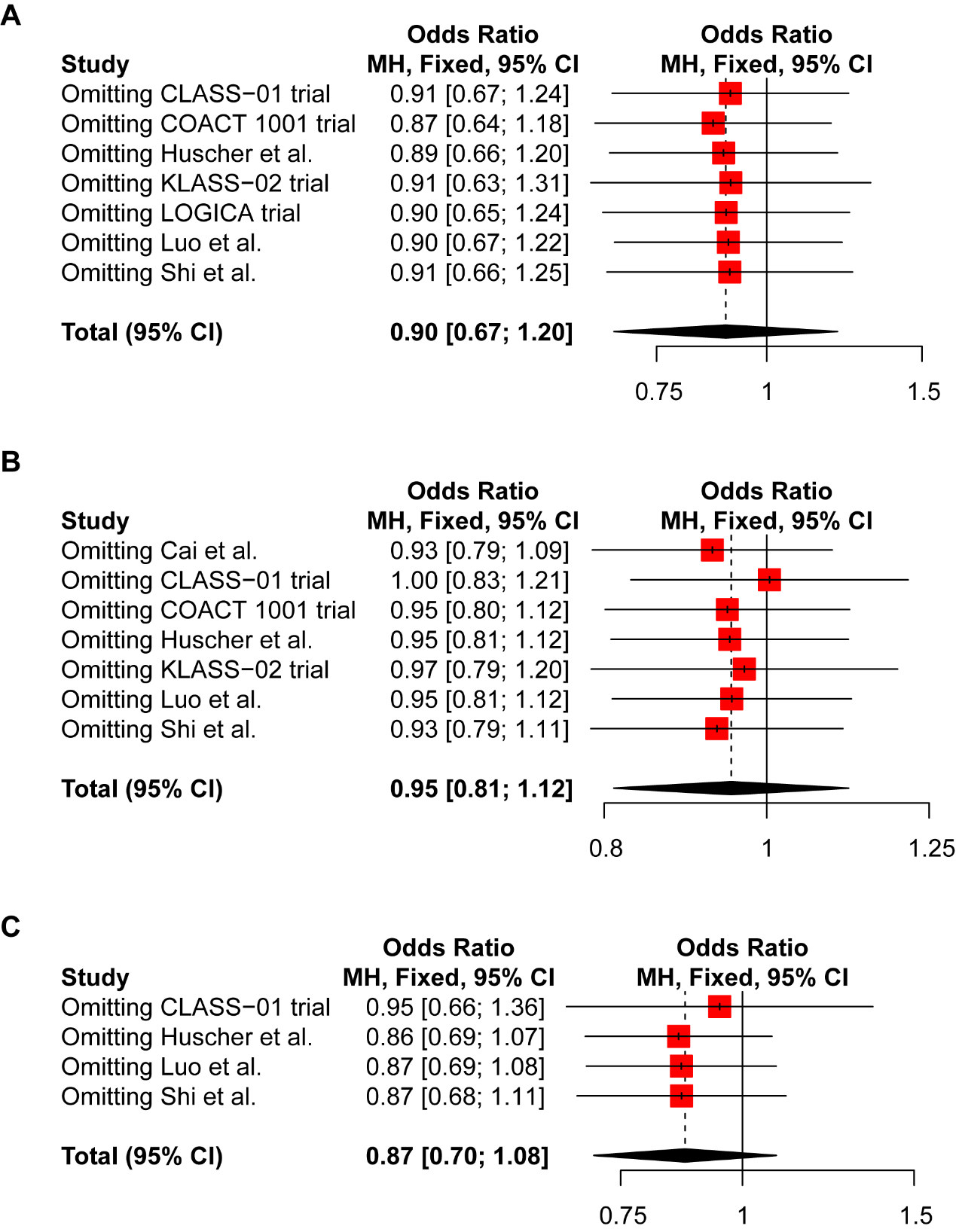


Figure 4: Sensitivity analysis for (a) 1-year survival rate; (b) 3-year survival rate; (c) 5-year survival rate
